# Evidence for human milk as a biological system and recommendations for study design—a report from “Breastmilk Ecology: Genesis of Infant Nutrition (BEGIN)” Working Group 4

**DOI:** 10.1016/j.ajcnut.2022.12.021

**Published:** 2023-05-10

**Authors:** Sharon M. Donovan, Nima Aghaeepour, Aline Andres, Meghan B. Azad, Martin Becker, Susan E. Carlson, Kirsi M. Järvinen, Weili Lin, Bo Lönnerdal, Carolyn M. Slupsky, Alison L. Steiber, Daniel J. Raiten

**Affiliations:** 1 Department of Food Science and Human Nutrition, University of Illinois, Urbana-Champaign, IL, USA; 2 Department of Anesthesiology, Pain, and Perioperative Medicine, Department of Pediatrics, and Department of Biomedical Data Sciences, School of Medicine, Stanford University, Stanford, CA, USA; 3 Arkansas Children’s Nutrition Center and Department of Pediatrics, University of Arkansas for Medical Sciences, Little Rock, AR, USA; 4 Manitoba Interdisciplinary Lactation Centre (MILC), Children’s Hospital Research Institute of Manitoba, Department of Pediatrics and Child Health and Department of Immunology, University of Manitoba, Winnipeg, Manitoba, Canada; 5 Department of Dietetics and Nutrition, University of Kansas Medical Center, Kansas City, KS, USA; 6 Department of Pediatrics, Division of Allergy and Immunology and Center for Food Allergy, University of Rochester Medical Center, New York, NY, USA; 7 Biomedical Research Imaging Center and Department of Radiology, University of North Carolina at Chapel Hill, Chapel Hill, NC, USA; 8 Department of Nutrition, University of California, Davis, CA, USA; 9 Department of Food Science and Technology, University of California, Davis, CA, USA; 10 Academy of Nutrition and Dietetics, Chicago IL, USA; 11 Pediatric Growth and Nutrition Branch, *Eunice Kennedy Shriver* National Institute of Child Health and Human Development, National Institutes of Health, Bethesda, MD, USA

**Keywords:** human milk, infant development, immune, microbiome, systems biology

## Abstract

Human milk contains all of the essential nutrients required by the infant within a complex matrix that enhances the bioavailability of many of those nutrients. In addition, human milk is a source of bioactive components, living cells and microbes that facilitate the transition to life outside the womb. Our ability to fully appreciate the importance of this matrix relies on the recognition of short- and long-term health benefits and, as highlighted in previous sections of this supplement, its ecology (i.e., interactions among the lactating parent and breastfed infant as well as within the context of the human milk matrix itself). Designing and interpreting studies to address this complexity depends on the availability of new tools and technologies that account for such complexity. Past efforts have often compared human milk to infant formula, which has provided some insight into the bioactivity of human milk, as a whole, or of individual milk components supplemented with formula. However, this experimental approach cannot capture the contributions of the individual components to the human milk ecology, the interaction between these components within the human milk matrix, or the significance of the matrix itself to enhance human milk bioactivity on outcomes of interest. This paper presents approaches to explore human milk as a biological system and the functional implications of that system and its components. Specifically, we discuss study design and data collection considerations and how emerging analytical technologies, bioinformatics, and systems biology approaches could be applied to advance our understanding of this critical aspect of human biology.

## Introduction

The “Breastmilk Ecology: Genesis of Infant Nutrition (BEGIN)” Project was designed to: *1*) examine the ecology of human milk, based on the supposition that human milk represents a complex biological system that interacts with both the internal biology and health of the lactating person, the human milk matrix, and the impact on the breastfed (BF) infant and external (social, behavioral, cultural, and physical) environments (see Text Box 1 for Core Concepts and Terms); *2*) explore the functional implications of this ecology for both the biological parent and their infant; and *3*) explore ways in which this emerging knowledge can be studied and expanded via a targeted research agenda and translated to support the community’s efforts to ensure safe, efficacious, equitable, and context-specific infant feeding practices in the United States and globally. The matrix of human milk refers to the nutrient and nonnutrient components of foods and their molecular relationships to each other (USDA).

Text Box 1 **Core concepts and terms**

• In the context of this paper, “ecology” is defined as a complex biological system and its interactions with its environment. In this case, the complex system is human milk composition and its inherent biology, and the environment consists of parental and infant inputs and the influence of their respective internal and external environments.
• With due recognition of the need to be observant of issues of gender identity/neutrality, and to improve precision, to the extent possible, for the purposes of the papers described herein, we will use gender neutral terminology where appropriate (e.g., lactating parent/person, etc.), to reflect the reality that not all who lactate identify as female. The term “lactating parent” respects and recognizes those who may have been born female but do not identify as such as well as other gender-relevant contingencies. In situations where reporting primary data (studies/analyses), we will refer to the population as specified (e.g., “the study evaluated 250 lactating mothers”). Moreover, rather than using terms such as “maternal” or “maternal milk,” we will use the terms such as “birthing parent” throughout the report as appropriate as they accurately reflect the biological nature of the birthing parent–infant dyad.
• “Human milk” refers to milk produced by lactating parents and includes both: *1*) breastmilk produced by a parent for their infant and fed directly to infants via the breast or expressed by the lactating parent and then fed to the infant; and *2*) donor/banked human milk produced by lactating persons that is either donated to human milk banks or fed to infants other than their own child.

Alt-text: Text Box 1

The overarching conceptual framework and description of the Project is presented in the BEGIN Executive Summary, the first of 6 manuscripts of this supplement. The subsequent manuscripts in this supplement present the findings of the individual thematic BEGIN Working Groups (WGs) as a continuum of thought that reflects a larger conceptual view of how we can move this important research and public health agenda forward [[1], [2], [3], [4]]. Specifically, the BEGIN Project was accomplished by forming 5 thematic WGs charged with addressing the following themes: *1*) parental factors affecting human milk production and composition; *2*) the components of human milk and the interactions of those components within this complex biological system; *3*) infant factors affecting the matrix, emphasizing the bidirectional relationships associated with the breastfeeding dyad; *4*) the application of existing and new technologies and methodologies to study human milk as a complex biological system; and *5*) approaches to translation and implementation of new knowledge to support safe and efficacious infant feeding practices. This paper represents the results of the deliberations of WG 4.

The key concept underlying the BEGIN Project is that human milk exists as a biological system that reflects both internal (lactating parent) and external (infant and environmental) influences [5,6]. To advance our understanding of human milk as a biological system, the BEGIN WGs considered interactions within and between each component of the lactating parent–milk–infant triad [[1], [2], [3], [4]]. However, no studies have attempted to integrate datasets across the triad to determine how factors such as genetics of the lactating parent or the physical/social/behavioral environment of either the lactating parent and/or infant shape the triadic interactions.

Many challenges exist in delineating the functional implications of human milk as a biological system, including the inherent complexity of human milk, the limited availability of noninvasive techniques suitable for use in infants, and the historical lack of application of systems biology approaches to human milk and lactation research. Studies of human milk bioactivity have largely focused on breastfeeding practices (e.g., longer versus shorter duration of breastfeeding), single human milk components, or a limited number of outcomes. These studies fail to account for the full biological potential of the human milk matrix, as viewed within the context of the “nourish, protect, and communicate” paradigm outlined by WG 2 [2].

The task of BEGIN WG 4 was to identify approaches to integrate the interactions of the lactating parent–human milk–infant triad. WG4 explored how emerging analytical technologies, bioinformatics and systems biology approaches could be used to advance our understanding of this critical aspect of human biology (Text Box 2).

Text Box 2 **BEGIN WG 4 report outline**

Section 1 Human milk and infant feeding on outcomes of term infants
Section 2 Human milk components, alone or in combination on outcomes of term infants
Section 3 Experimental framework and analytical approaches to study human milk as a complex biological system in term infants

Alt-text: Text Box 2

## Section 1: Human Milk and Infant Feeding on Outcomes of Term Infants

In this section, we summarize key findings and limitations of available evidence linking human milk composition and feeding practices to infant outcomes, which were selected based on the potential for long-term programming by dietary intake during early postnatal life (see Text Box 3). A common approach has been to compare human milk–fed to formula-fed (FF) infants. Although these studies have informed how human milk benefits infant development, it is not known whether the differences in outcomes between BF and FF infants result from nutritional components that are present (e.g., bovine milk proteins) or immunologic factors impacting signaling mechanisms (e.g., bioactive components) being present, absent, or available in different levels in infant formula compared with human milk.

Text Box 3 **Infant functional outcomes of interest for the study of human milk**

• Physical growth and body composition
• Endocrine development
• Neurocognitive development
• Intestinal development and microbiome composition
• Immune system development and tolerance
• Metabolome

Alt-text: Text Box 3

Furthermore, the forms and concentrations of nutrients and the matrix in which they exist differ markedly between human milk and infant formula. Three main experimental approaches have been used to study how variability of one or more components *within the matrix* of human milk composition impacts infant outcomes. First, evaluating dietary interventions to the lactating parent to modify one or more components within human milk. Second, studying triads experiencing environmental conditions that could modify human milk composition. Lastly, investigating genetic differences in the lactating parent or offspring, for example secretor status and human milk oligosaccharide (HMO) content and composition.

In Section 1, we summarize the evidence regarding human milk feeding and its impact on key functional outcomes (Text Box 3). Growth and physical development are highlighted, as studies have been conducted to examine differences in exposure to human milk (e.g., exclusivity and duration) as well as effects of nutrient supplementation and genetics of the lactating parent on infant growth and body composition. Other functional domains are briefly introduced in the text with key observations, proposed mechanisms of action and limitations summarized in Table 1 [[7], [8], [9], [10], [11], [12], [13], [14], [15], [16], [17], [18], [19], [20], [21], [22], [23], [24], [25], [26], [27], [28], [29], [30], [31], [32], [33], [34], [35], [36], [37], [38], [39], [40], [41], [42], [43], [44], [45], [46], [47], [48], [49], [50]].

**TABLE 1:** Multifunctional effects of human milk on infant and child outcomes.

Functional System		Findings [reference]
**Neurocognitive Development**	Observations	• A meta-analysis of studies involving >12,000 children reported that those breastfed (BF) for ≤6 mos versus >6 mos had 1.04- and 1.06-fold higher scores on intelligence tests than those never BF, respectively [7]. • Associations between variable concentrations of LC-PUFA (8–11) and choline and lutein [12] in human milk and IQ have been shown. • Synergistic associations of higher levels of both choline and DHA in human milk with better recognition memory in infants [12]. • Meta-analyses of RCT of LC-PUFA supplementation have not shown an impact of consumption of fish oil or DHA/EPA supplements in breastfeeding females on cognitive performance of their children [13, 14]
	Proposed Mechanisms or Associations with Human Milk Components	• Improved myelination by 2 y of age in BF children, including networks associated with a broad array of cognitive and behavioral skills [15] • Sialylated molecules, including gangliosides and sialoproteins, are present in the frontal cortex of infants [16] • In BF, but not in FF infants, ganglioside-bound sialic acid was correlated with ganglioside ceramide DHA and total n-3 fatty acid, suggesting potential interactions between human milk LC-PUFA and HMOs. [16]
	Limitations	• Small sample size studies, short-term studies with short follow-up. lack of diversity, lack of information on maternal and child genetic polymorphisms in LC-PUFA synthesis, lack of report of sociodemographic factors [13,14,17]. • Within human milk component interactions have not been explored (HMO to other components, HMO-HMO interactions).
**Endocrine Development**	Observations	• Type 2 Diabetes: BF reduced risk by 33% [18]. • Type 1 Diabetes: • Exclusive BF for >2 wk was associated with a 14% lower risk than in a shorter duration and/or a lack of BF [[19], [20], [21]].
	Proposed Mechanisms or Associations with Human Milk Components	• Breastfeeding is associated with lower preprandial serum glucose and insulin concentrations than in formula-feeding [22]. • Type 2 Diabetes: Postulated effects on appetite regulation, reduced weight gain during infancy, and/or nutrients in human milk that promote energy balance, independently of child or adult BMI [23]. • Type 1 Diabetes: • Lower gut permeability in BF than FF infants [24] • Immunomodulatory substances, such as lactoferrin, lysozyme, and secretory immunoglobin A (sIgA), as well as macrophages that affect the function of T- and B cells [25] • Other human milk components have been suggested, but mechanistic evidence in humans is lacking: SCFA [26,27] human milk-derived opioid peptides β-casomorphins [28], HMOs [29], fucosyltransferase-2 nonsecretor status [30], miRNA content of human milk exosomes [31]
	Limitations	• Type 1 Diabetes: Unknown whether human milk offers protection in high-risk populations with genetic predisposition [32] • Lack of well controlled studies with longitudinal outcomes and comprehensive information on human milk ecology and composition
**Intestinal Development**	Observations	• Endoscopic biopsies from healthy infants demonstrated 30% greater crypt length in FF than BF infants [33]. • Intestinal permeability *in vivo* was higher in FF than BF infants [24,34].
	Proposed Mechanisms or Associations with Human Milk Components	• Pathways regulating stem cell proliferation, differentiation, and migration, as well as barrier function and immune response were differentially expressed in exfoliated epithelial cells of BF vs. FF infants [35]. • Diet can affect, via gut colonization and cross talk with host epithelial cell, expression of genes associated with the innate immune system in infants [36].
	Limitations	• Limited sample sizes (4-10 infants per group) • Lack of longitudinal data on diet and infant intestinal development • Need to delineate developmental from human milk effects (e.g., term vs. preterm) • Need to correlate gene expression with function and developmental outcomes
**Immune Development**	Observations	Immune cells: • CD4+ T cells: No consensus for an effect of BF [[37], [38], [39]] • CD8+ T cells: Longer BF was associated with increased CD8 T cell memory, but not memory B cell numbers, in the first 6 mo [39]. • Natural killer cells: Higher in BF than formula-fed infants at 6 mo of age [37]. • Tregs: Proportion of Tregs increased nearly two-fold between birth and 3 wk of age in BF then FF infants [40]. • IgA and IgG secreting B cells: Higher in FF than BF infants [41]. Cytokines • Serum proinflammatory TNF-α and IL-2 were higher, while TGF-β2 was lower, in FF than BF infants in the first year [42]. • BF neonates showed a specific and Treg-dependent reduction in proliferative T-cell responses to noninherited maternal antigens, associated with a reduction in inflammatory cytokine production [40]. IgA: • Earlier and greater IgA production in nasal and saliva samples from BF compared with FF infants in the first few days of life [43], but not at 7 wks [44]. • Inconsistent finding on salivary IgA, IgM, and IgG between 3 and 6 mos [45,46]. Thymic size and GALT: • Breastfeeding increases thymic size compared with formula-feeding [47]. There are no human studies on the effect of breastfeeding or breast milk on the development of GALT due to lack of access to human tissues.
	Proposed Mechanisms or Associations with Human Milk Components	• Higher IgA and IgG secreting B cells in FF possibly due to higher antigenic exposure [41]. • Human milk TGFβ may induce IgA production in infants [48]. • IgA production may be induced by *Bifidobacterium* species (enriched in BF) [19,49]. • Human milk IL-7 content, an important factor for lymphocyte development, was correlated with thymic size [50,51]. • Thymic size was also correlated with the number of CD8+ T cells, which are increased in BF infants [39].
	Limitations	• Only observational studies. • Small sample sizes and limitations in the detection of memory T cells. • Unknown if mechanisms underlying higher proinflammatory cytokines in formula-fed infants are direct effects of human milk components or are mediated through gut microbiota. • Discrepancies in salivary IgA levels may depend on antigenic exposure and infant gut microbiome composition. • There are no human infant studies on the effect of BF or human milk on the development of GALT, due to lack of access to such human tissues.
**Clinical Immune Outcomes**	Observations	Food Allergy: • Protective effect of exclusive breastfeeding against cow’s milk allergy in early childhood among *high-risk* infants is inconsistent [52,53]. Other Atopic Diseases: • Moderate evidence for a protective effect of human milk consumption against asthma in childhood, limited evidence to indicate no association between human milk consumption and atopic dermatitis in childhood, and inconclusive evidence to suggest a relationship between human milk consumption and atopic dermatitis from 0-24 mo of age [20]. • No association between duration of human milk consumption and allergic rhinitis in childhood [20]. • Evidence is insufficient to suggest any relationships between human milk consumption and asthma, atopic dermatitis, or allergic rhinitis during adolescence or adulthood, or between human milk consumption and food allergy at any life stage [53]. Gut Inflammatory Diseases: • Inconclusive evidence of human milk consumption on celiac disease [54]. • Limited, but consistent, case-control evidence suggests that shorter versus longer durations of any human milk feeding are associated with higher risk of IBD [20].
	Proposed Mechanisms or Associations with Human Milk Components	• Higher concentrations of total IgA and casein-specific IgA in human milk have been associated with protection against cow’s milk allergy [55,56]. • TGFβ is the most well-studied human milk cytokine in connection with infant atopic outcomes; however, a meta-analysis found no association between TGFβ in human milk and allergic outcomes [57]. • High levels of some human milk cytokines (e.g., IL-1β, IL-6, IL-10, and TGFβ) are associated with protection against food allergic disease [58], and human milk IL-6 and IGF-I may play a role in oral tolerance [59]. • Effects of HMO are inconsistent with regard to atopic eczema, cow’s milk allergy, asthma and eczema [[60], [61], [62]].
	Limitations	• High heterogeneity and low-quality evidence, lack of adequate statistical power to assess the impact of BF [53].
**Serum and Fecal Metabolomes**	Observations	• BF infants have higher fatty acid metabolism compared with formula-fed infants, as shown by higher levels of free fatty acids, lysophosphatidylcholines, and long-chain acylcarnitines, as well as increased markers of β-oxidation in serum [63]. • FF infants have higher levels of circulating amino acids and amino acid degradation products in serum than BF [[64], [65], [66], [67], [68], [69], [70], [71], [72]]. • Higher levels of microbial degradation products of protein in feces of FF than BF infants [63,66,70,73]. • BF infants introduced to complementary feeding before 6 mos had higher serum BCAA at 12 mo of age than to BF infants who were exclusively BF to 6 mo of age [63,64].
	Proposed Mechanisms or Associations with Human Milk Components	• Differences in dietary composition between human milk and formula • Nutrients in human milk are packaged differently within the human milk matrix (e.g., fats with the MFGM)
	Limitations	• Further studies on human milk composition, including the human milk matrix, and nutrient absorption could determine if changes in human milk composition over time, or even during the day, alter the degree to which human milk nutrients are metabolized and absorbed • Few studies have examined the timing of the introduction of complementary feeding on metabolism • Limited evidence on interactions between diet, microbiome and metabolome and outcomes, such as growth
**Gut Microbiome**	Observations	• Gut microbiota at 12 mos differed compared with those weaned from human milk before 6 mo of age [74]. • BF infants supplemented with formula where BF was associated with higher *B. breve* and *B. bifidum*; cessation of BF resulted in faster maturation of the gut microbiome (Firmicutes) [75]. • Richness and diversity of microbiota were highest in infants who were not BF, lower in partially BF infants, and lowest in exclusively BF infants. Increasing exclusivity of breastfeeding was associated with greater relative abundance of *Bifidobacteriaceae* and *Enterobacteriaceae* and lower relative abundance of *Lachnospiraceae, Veillonellaceae*, and *Ruminococcacae* [74].
	Proposed Mechanisms or Associations with Human Milk Components	• Glycans cleaved from human milk proteins by microbial glycohydrolases [76] and peptides produced by proteolytic digestion *in vivo* [77] are bifidogenic • HMOs act as prebiotics and shape the infant microbiome [78]. • *B. longum* subsp. *infantis*, directly internalize intact HMOs by specific transporters and degrade them intracellularly [79], which allows it to outcompete other *Bifidobacterium* species/strains (*B. bifidum* and some *B. longum* strains) that use extracellular glycosidase(s) • *Bifidobacterium* populations increase more rapidly and are more abundant in infants fed by secretor mothers than those fed by nonsecretor mothers and specific *Bifidobacterium* strains that can use 2′FL are enriched in the stools of the infants receiving human milk of secretors vs. nonsecretors [80,81,82]. • Fucosylated α1-2 oligosaccharides are degraded by some *Bacteroides* (particularly *B. fragilis*) and Akkermansia (*A. muciniphila* MucT) [83], which are commonly present in the infant gut [75]. • Secretor status interacts with route of delivery; infants born by CS who were fed secretor human milk had a less dysbiotic gut microbiota compared with vaginally-delivered infants than did CS infants who received nonsecretor human milk [84]. • Human milk contains a milk microbiota, which has been implicated in seeding the infant microbiota [85].
	Limitations	• Data on microbiome composition of body sites besides fecal are limited • Most studies are limited to 16S rRNA analysis, with small sample sizes • Data on Archaea, viruses and fungi in human milk and infant stool are limited • HMO diversity and human milk microbiota composition and metabolic function are influenced by milk composition environmental factors, genetics, geographical location, and other factors [86,87], including differences based on secretor status [85,88,89], which are often not documented. • Low biomass samples, such as human milk may yield spurious results due to environmental contamination • 16s rRNA signatures do not reflect viable human milk microbes [90] • Few studies addressed microbiome function (e.g., metagenomics and metabolomics) and human milk matrix • Studies on BF and host-microbe interactions and long-term outcomes are limited

BF, breastfeeding; CS, Cesarean section; 2′FL, 2′-fucosyllactose; FF, formula-feeding; GALT, gut-associated lymphoid tissue; HMO, human milk oligosaccharide; IBD, inflammatory bowel disease; SCFA, short-chain fatty acids; Tregs, T-regulatory cells; TGFβ, transforming growth factor-beta.

### Physical growth and body composition

Several recent systematic reviews summarize the evidence linking human milk feeding and growth [[91], [92], [93]]. Most studies reported outcomes on linear or body weight gain compared with reference standards, and a few studies report longitudinal changes in body composition [91,92,[94], [95], [96]]. Limitations of existing evidence on whether or how breastfeeding and human milk affect growth outcomes are highlighted in Text Box 4. In addition, none addressed any of the aspects of the human milk ecology.

Text Box 4 **Limitations of studies investigating infant feeding practices and growth outcomes**

• Heterogeneity in outcomes and definitions of infant feeding practices (e.g., feeding at the breast vs bottle, other human milk source [donor milk], exclusivity)
• Inconsistent reporting of nutrient composition and volume of human milk consumed
• Inconsistent growth outcomes reported (body weight, linear growth, body composition, *z*-scores, weight velocity), and
• Majority of studies are observational, which may introduce bias

Alt-text: Text Box 4

#### Exclusivity and duration of human milk exposure

Current evidence associates longer duration of exclusive or partial breastfeeding with slower infant growth rates in term infants than in mixed feeding or formula feeding in developed countries [93]. A recent systematic review of predominantly prospective observational trials concluded that there was insufficient evidence to determine if duration of exclusive breastfeeding reduced risk for obesity [97]; however, moderate evidence supports that ever vs never breastfeeding, particularly for longer than 6 mo, is associated with a reduced risk of overweight and obesity at ages 2 y and older [91,95]. Considering that observational studies are prone to bias due to confounding, a separate analysis was conducted of studies that used more rigorous study designs, including 4 US observational cohorts that included sibling-pair analysis along with the Promotion of Breastfeeding Intervention Trial (PROBIT) in the Republic of Belarus [97,98]. This analysis found that the association between ever vs. never breastfeeding and risk of childhood overweight and obesity was not significant [97]. Thus, future longitudinal studies are needed involving siblings to assess associations and to provide heightened confidence in causal findings.

To date, PROBIT is the sole cluster randomized study evaluating the effects on multiple outcomes of a breastfeeding promotion program that led to a longer duration of breastfeeding in the intervention group compared with the control group [98]. In addition, health outcomes through adolescence have been reported. As such, PROBIT can be viewed as a model for future study design and expanded to include broader aspects of the internal and external ecologies described in Section 3. Although the PROBIT trial is commonly referenced for breastfeeding outcomes, some limitations have been noted in which Belarus may not be representative of other countries. These include families having access to good basic health services, 3-year maternity leaves with little use of daycare, 95% breastfeeding initiation rate, a well-educated population, and that non-BF infants were excluded from the trial [99].

Analysis of data from the ongoing Canadian Healthy Infant Longitudinal Development (CHILD) birth cohort study revealed an inverse association between breastfeeding and weight gain velocity/BMI in a dose-dependent manner, which was diminished when human milk was fed from a bottle and when human milk was combined with formula supplementation [74,100]. This study investigated important aspects of how infants are fed but has not fully addressed human milk ecology.

Body composition outcomes with breastfeeding are less well studied [101], and only 1 meta-analysis of 11 studies demonstrated that never being BF was associated with altered body composition in infancy, with higher body fat mass in early infancy and lower body fat mass in the second year of life [91]. Hormone content of human milk (leptin, ghrelin, insulin-like growth factor-I [IGF-I], adiponectin, and insulin) has been explored in relationship to infant hunger, fat deposition, and adipose tissue metabolism [102]. To date, no consistent effects were observed, which may be attributed to the reliance on gross anthropometric measures and cross-sectional studies of short duration that did not include early time points. Moreover, traditional component-based studies have not recognized the potential importance of integrating components of human milk while considering its matrix and ecology.

#### Dietary interventions to the lactating parent

A meta-analysis of data from low- and middle-income countries reported that supplementation with multiple micronutrients during lactation did not improve infant length, stunting, or head circumference [103]. Similarly, a Cochrane systematic review found that long-chain polyunsaturated fatty acid (LC-PUFA) supplementation to lactating parents in high-income countries (HIC) yielded no differences in children’s growth [13]. The absence of associations raises questions about the type of supplemented nutrients, their concentration, and the dynamic human milk ecology that may have influenced human milk volume intake [104,105]. More comprehensive study designs accounting for the complexity of human milk composition and its matrix, human milk volume intake and precise longitudinal outcomes could enhance our understanding of the associations between maternal dietary intake and infant development.

#### Genetic differences affecting human milk composition

The most prominent example of the examination of genetic variations on human milk composition have focused on variation in genes for encoding for secretor and Lewis phenotypes influencing HMO content and composition [106]. Associations between HMO content and growth of BF infants have been inconsistent. Variations in concentrations of 2′-fucosyllactose (2′FL), difucosyllactose, lacto-N-neotetraose (LNnT) and lacto-N-fucopentaose I have been associated with weight velocity, height-for-age Z-scores and body composition in some studies but not others [107,108]. Recently, significant associations between human milk bacteria and HMO intakes and concentrations and infant anthropometry, fat-free mass, and adiposity were shown [109]. When data were stratified based on maternal secretor status, some relationships were found among infants born to secretor vs nonsecretor mothers [109]. While these studies have examined associations with all HMOs in the human milk sample, which is more robust than single component associations, they do not encompass the full human milk ecology and matrix complexity and, thus, the conflicting results should be viewed with caution.

In summary, future prospective cohort studies should incorporate robust study design, with standardized and frequent measurements of growth and body composition, measurement of human milk composition and intake, and documentation of pertinent parental and infant co-variates to uncover underlying mechanisms whereby human milk and/or breastfeeding influence infant growth. The extant data is limited in assessing the impact of exclusivity or duration of human milk feeding, parental supplementation or that of genetic differences on the human milk matrix.

#### Neurocognitive and functional development

Neurodevelopment is exquisitely sensitive to early life dietary and environmental exposures and has been an active area of research [110]. Some [111,112], but not all [113,114], observational studies of human milk–fed term infants have reported higher cognitive outcomes than in FF term infants, without associating benefit to any particular component of the human milk matrix (Table 1). Deoni et al [15] found that exclusive breastfeeding for at least 3 mo was associated with improved myelination diffusely throughout the brain by 2 y of age, which persisted through early childhood. In a meta-analysis, breastfeeding compared with formula-feeding was associated with an increased intelligence quotient (IQ) of ∼3.5 points in childhood and adolescence and 2.19 points after adjustment for maternal IQ [115]. In terms of breastfeeding duration, a meta-analysis of studies involving >12 000 children showed that those BF for either ≤6 mo or >6 mo had higher scores on intelligence tests than those never BF [7].

The effect of LC-PUFA supplementation in the lactating parent, either as fish oil or as purified forms of DHA, EPA, and/or AA on infant neurocognitive development has been investigated. Meta-analyses of randomized control trials have not shown associations between the consumption of fish oil or DHA/EPA supplements in breastfeeding females and the cognitive performance of their children [13,14]. A systematic review, conducted as part of the 2020 Dietary Guidelines, found there was insufficient evidence to evaluate the effects of omega-3 fatty acid supplementation during pregnancy and/or lactation on other infant developmental outcomes [116]. These reviews identified the need for additional studies with larger and more diverse sample sizes and inclusion of information on maternal and child genetic polymorphisms in LC-PUFA synthesis and sociodemographic factors.

#### Endocrine development

Few studies have investigated how human milk feeding affects endocrine development, other than for risk of type 1 and type 2 diabetes. Updated systematic reviews and meta-analyses suggest that breastfeeding reduces the odds for the development of type 2 diabetes by 33% [18]. For type 1 diabetes, exclusive breastfeeding for >2 wk reduces risk by 14%, compared with a shorter duration [19,20] and/or a lack of breastfeeding [21]. Potential mechanisms and limitations of the current evidence are summarized in Table 1 and include a lack of assessment of the human milk matrix.

#### Intestinal development

The intestinal tract is functionally immature and immunologically naïve at birth [116] and undergoes marked structural and functional adaptation in response to feeding [24,117]. The trophic response to human milk exceeds that of formula, suggesting a unique contribution of human milk constituents and importantly, the human milk matrix, are important in this response [118,119]. Most studies on the impact of feeding have been conducted in preclinical animal models [120] or preterm infants [121] due to the availability of tissue samples or aspirates. However, measuring the transcriptome of exfoliated epithelial cells has allowed for noninvasive interrogation of intestinal gene expression in term infants [35], affording a noninvasive approach to longitudinally assess the impact of diet on gut health and function in BF infants. Observations of the effect of human milk on intestinal development of infants and potential mechanisms and limitations of the current evidence, which include a lack of dissection of the human milk matrix effect, are summarized in Table 1.

#### Immune development and clinical immune outcomes

The immune system is also immature at birth as evidenced by incomplete physical and chemical barriers, poor innate effector cell function, limited and delayed secretory immunoglobulin A (IgA) production, underdeveloped complement cascade function, and insufficient anti-inflammatory mechanisms of the intestinal and respiratory tracts [122]. As reviewed by Dawod et al [123], human milk contains many immune components that are purported to facilitate the transition of the infant to extrauterine life. Microbial colonization of the intestinal tract in early life also plays a key role in stimulating the development of mucosal immunity and long-term programming of the adaptive immune system [49,[124], [125], [126]]. Surprisingly little is known of how human milk influences the developmental ontogeny of peripheral blood immune cells, cytokines or gut-associated lymphoid tissue in human infants [[37], [38], [39], [40], [41], [42], [43], [44], [45], [46], [47],^127^] (Table 1).

The impact of human milk consumption on clinical immune outcomes (e.g., atopic diseases and GI inflammatory diseases) is summarized in Table 1. To date, only PROBIT showed that increased duration and exclusivity of breastfeeding was associated with decreased risk of atopic eczema in the first year of life [97]; however, follow-up at 6.5 y did not support a protective effect of prolonged or exclusive breastfeeding on allergy or asthma [128].

Most of the available evidence describing relationships between shorter or longer periods of exposure to human milk, or naturally occurring low versus high levels of human milk components, and outcomes such as IgE development, humoral immunity, and food allergy is limited to observational studies in humans, which are suitable only for suggesting association, not for determining causation. In addition, the evidence is difficult to interpret due to application of varying definitions of breastfeeding and allergic disease, potential for reverse causality, insufficient power for or lack of reporting of specific atopic disease outcomes, and the potential confounding effect of gene-environment interactions.

#### Serum and fecal metabolome

Metabolomics has emerged as an important tool to investigate how infant diet (human milk vs. infant formula with or without added bioactive components) impacts serum, fecal and urinary metabolites (Table 1). Human milk–fed infants have higher concentrations of fatty acid metabolites [63] than FF infants, who have higher levels of amino acids and products of amino acid degradation [[64], [65], [66], [67], [68], [69], [70], [71]], suggesting that the composition of human milk enhances fat-based metabolism [64,69].

#### Fecal microbiome composition

There are multiple microbial ecologies that influence human health and development. While less is known about several of these microbiomes (oral, epithelial, vaginal etc.), emerging evidence indicates a significant role of infant feeding in the development and health of the gut microbiome. The gut microbiome is established over the first 2 to 3 y of life through sequential phases that are influenced by numerous factors, most notably route of delivery and form of nutrition [129,130]. Microbial colonization of the gastrointestinal tract is essential for programming of infant immune, neurocognitive, and intestinal development [49,123,131]. The process can be viewed through the lens of ‘seeding, feeding, and weeding’, in which route of delivery provides the first exposure to environmental bacteria, which are subsequently shaped by nutrition and antibiotic exposure, among other environmental factors, including geographical location and household exposures [132,133]. The microbiota of infants born by Cesarean section (CS) who were exclusively BF was more similar to vaginally-delivered infants than FF, CS-delivered infants [134]. Thus, when assessing the impact of human milk on the infant microbiota, it is essential to collect metadata related to both birthing parent and infant exposures.

Large longitudinal cohort studies show that breastfeeding duration and exclusivity influence infant gut microbiome composition and function, with exclusive human milk feeding establishing a less diverse and different microbiota that can be disrupted by formula feeding, particularly in the early postpartum period [74,75]. In addition, HMOs act as prebiotics and shape the infant microbiome [78], thus, the genetics (secretor status) of the lactating parent can influence microbial colonization of the recipient infant by modifying the composition of human milk [80]. Observations of the effect of human milk on infant gut microbiome development of term infants and potential mechanisms and limitations of the current evidence are summarized in Table 1.

### Conclusions

Existing clinical and epidemiological evidence support improved health and developmental outcomes for infants fed human milk. To date, there is limited research that has explored the full ecology of human milk including critical aspects of the triadic relationships highlighted by WG 1-3 [[1], [2], [3]], and their importance in fully understanding the impact of human milk on infant health and development. Moreover, most studies have focused on a single outcome (e.g., growth, immune development, microbiome composition) rather than taking a holistic view of human milk as a developmental modulator. Although it is important that we apply a systems biology approach to study human milk as a matrix, it is clear that we also need to consider the systems biology within the recipient infant. For example, there is an ever-growing appreciation of the interactions between the microbiota and the development of other organ systems, including gut, immune, and neurocognition. To gain a better understanding of the functionality of human milk components, alone or in combination, the next section will briefly review findings of randomized controlled trials (RCTs) conducted in human infants.

## Section 2. Human Milk Components, Alone or In Combination on Outcomes of Term Infants

In addition to the types of studies reviewed in Section 1 that have explored the impact of human milk on specific functional outcomes, considerable effort has gone into the study of specific constituents of human milk on infant health and development. While these studies have rarely explored the intersection of the human milk ecology and the triad, they have provided some potential avenues for pursuit of a deeper understanding of that intersection. The following brief review explores some examples of milk constituents and the most common approaches that have been used to evaluate their impact on infant health and development.

A common approach to the exploration of the impact of milk constituents has been the use of analogs of human milk bioactives isolated from bovine milk or synthesized to study their physiological functions in preclinical models and human infants. Among the most studied are HMO, milk fat globule membrane (MFGM), osteopontin (OPN) and LC-PUFA [135]. Summarized in this section and in Table 2 are findings of RCTs conducted in infants that evaluated the bioactivities of these milk components. These case studies highlight the multifunctional activities of individual milk components. Notably these studies explore these relationships outside of the biological matrix of human milk, which should be considered when assessing human milk as a biological system.

**TABLE 2:** Multifunctional bioactivities of isolated milk components supplemented to infant formula.

Milk Component and [concentration] (ref)	Functional Outcome					
	Growth/Tolerance	Infection	Immune Function	Neurocognitive	Microbiome	Serum Metabolites
**HMOs**						
• 2′FL [0.2 or 1.0 g/L] + galacto-oligosaccharides (GOS) to 2.4 g/L • 4 month intervention • BF reference [136,137]	Growth of 2′-FL supplemented infants not different than SF [136]	NR	• 2′FL reduced circulating pro-inflammatory cytokine concentrations and their secretion by isolated PBMC stimulated with RSV compared with SF; levels not different than [137]	NR	NR	• 2′FL detected in blood and urine of BF infants and those fed formula + 2′-FL [136]
• 2′FL [1.0 g/L] and LNnT [0.5 g/L] • 6 month intervention • Follow-up at 12 mos [138,139]	• HMO-supplemented infants had softer stool and fewer nighttime wake-ups at 2 mo of age [138]	• Reduced infectious episodes in the HMO-supplemented group [138] • Fewer parental reports of bronchitis through 4, 6, and 12 mo and LRTI through 12 mo [138] • Reduced parent-reported antipyretics use through 4 mo and antibiotics use through 6 and 12 mo [138]			• Microbial alpha and beta diversity of the HMO group was closer to that of the BF than control formula [139]	
• 5 HMO: LNT, 2′FL, 3′FL, 3′SL, 6′SL • Total HMO concentration of 5.75 g/L • 4 month intervention [141]	• No differences in growth or tolerance between infants fed SF or formula + 5 HMO					
**Lactoferrin**						
• Term infants fed formula with native bLF [102 mg/L] or added bLF [850 mg/L] for 12 mo [146]		• Reduction of URTI and wheezing (P < 0.05)				• Higher hematocrit levels at 9 mos (37.1% vs 35.4%; P < 0.05) in bLF-supplemented than CON
• Term infants fed formula or added bLF [38 mg mg/L] for 3 mo (105)		• Lower respiratory-related illnesses and occurrences of diarrhea-related illnesses BF and bLF groups than CON (P < 0.05)				
• Preterm, VLBW infants • Multicenter, randomized, double-blind, placebo-controlled trial • Orally dosed with bLF [100 mg/d, LF]; LGG [6×10^9^ CFU/d [151] bLF+LGG]; or placebo (CON) • Birth to 30 d of life [151, 152]	• No adverse effects or intolerances to treatment occurred [151]	• Incidence of ≥ stage 2 NEC and of death-and/or ≥ stage 2 NEC was lower in LF (p =0.055) and LF+LGG (p<0.001) vs. CON • Incidence of LOS due to bacterial or fungal infection was lower in LF (p=0.002) and LF+LGG (p<0.001) vs. CON (109)		•		
• Preterm, VLBW infants <32 wk GA • Multicenter (37 centers), randomized, double-blind, placebo-controlled trial • Orally dosed with bLF [150 mg/kg/d, LF]; or sucrose [CON] • ≤ 72 hours postpartum to 34 wk PMA [153]		• 316 (29%) of 1093 LF infants acquired a LOS vs 334 (31%) of 1089 CON infants • Risk ratio adjusted for minimization factors was 0.95 (95% CI 0.86-1.04; p=0·233).				
• Preterm infants weighing 500-2000 g • Multicenter (3 centers), randomized, double-blind, placebo-controlled trial • Orally dosed with bLF [200 mg/kg/d, LF]; or sucrose [CON] • 8 week intervention • Follow-up at 24 mo [154]	• Growth outcomes and rehospitalization rates during the 2-year follow-up were similar in both groups	• LOS or sepsis-associated death occurred in 22 LF infants (10.5%) vs 30 (14.6%) CON • No difference after adjusting for hospital and birth weight; hazard ratio 0.73 (95% CI, 0.42-1.26). • At 24 mo, LF infants had less bronchiolitis than CON (rate ratio, 0.34; 95% CI, 0.14-0.86).		• Mean age-adjusted normalized Mullen composite score at 24 mo was 83.3 ± 13.6 in the LF group vs. 82.6 ± 13.1 in CON (N.S.).		
• LF (0.6 g/L +MFGM (5 g/L) for 1 y study • Follow-up at 1.5 y [156]	• No difference in growth vs. CON	• Respiratory-associated adverse events and diarrhea were significantly lower for the MFGM + LF group through 1.5 y (p<0.05)		• Bayley II mean cognitive (+8.7), language (+12.3), and motor (+12.6) scores were higher (P< 0.001) for the MFGM + LF group vs. CON at 1 y • Differences no longer present at 1.5 y		
**MFGM**						
• MFGM protein fraction twice daily added to weaning food • 6- to 11-month-old infants [158]		• Global prevalence of diarrhea was 3.84% in MFGM vs 4.37% in CON (P < 0.05). • MFGM reduced episodes of bloody diarrhea by 46% (P = 0.025)				
• Formula with or without added MFGM fed for 4 mos • MFGM proteins constituted 4% (wt:wt) of the total protein content • ∼2-6 month-old infants • Follow-up at 12 mo • BF reference [63, 64, [159], [160], [161], [162], [163]]	• No effect of MFGM on growth or tolerance vs. CON [169] • At 6.5 y of age, no differences between MFGM and CON in weight, length, or head or abdominal circumference [163]	• Lower incidence of diarrhea, otitis media and anti-pyretic use vs. CON [159, 160]		• At 12 mo of age, Bayley cognitive score was higher (P = 0.008) in the MFGM (105.8 ± 9.2) than CON (101.8 ± 8.0), but was not different than BF (106.4 ± 9.5; P = 0.73) [159]. • At 6.5 y of age, no differences between MFGN and CON in any measure of cognitive or adaptive functioning [163]	• Little effect on fecal microbiome between MFGM and CON [63] • MFGM fed infants had a lower abundance of oral *Moraxella catarrhalis* than CON [161].	• MFGM reduced fecal lactate, succinate, amino acids and their derivatives vs. CON [63] • Infants fed MFGM had higher levels of fatty acid oxidation products in serum than CON [64] • Plasma lipidome of infants fed MFGM differed at 4 mo (SM and PCs) and 6 mos (SM, PCs, ceramides) vs. CON [162] • Erythrocyte SMs, PEs and PCs differed between MFGM and CON at 6 mo [162] • At 6.5 y of age, no differences between MFGM and CON plasma concentrations of homocysteine, lipids, insulin, or glucose [163]
**Osteopontin**						
• Double-blind RCT • Formula with 0 (CON), 65 (F65) or 130 (F130) mg/L bovine OPN for 6 mos • BF reference [165,167,168]	• Growth patterns, formula intake, sleep patterns and adverse events were similar in all formula-fed groups [167]	• Infants fed OPN had significantly fewer days with fever than CON [167]	• Infants fed OPN had serum TNF-α and higher IL-10 than CON (124) • Infants fed F130 had higher T-cell proportions than F0 or F65 [168]			• At 4 and 6 mo, plasma human OPN was higher in BF, F65, and F130 than CON [165] • Plasma bovine OPN in F130 was greater than F65 [165]
**LC-PUFA**						
• Double-blind RCT (DIAMOND STUDY) • Term infants, n=∼40 per group • Compared 4 formula containing 0.64% AA and either 0% (CON), 0.32%, 0.64% or 0.96% DHA for 12 mo [180]				• Any level of DHA compared with CON (0% DHA) improved: • Visual acuity in infants fed at 12 mo, but not 3 mo [180] • Cognitive development through 6 y of age [181] • Brain ERP responses and synchronization during a task requiring response inhibition at 5.5 y [182] • Brain structure, function and metabolism at 9 y [183]		
• RCT • Preterm infants <33 wk GA and 750-1800 g) • Compared CON formula with formula containing 0.26% DHA and 0.42% AA from either fish/fungal or egg/fish oil sources • Body composition measured by DXA [185]	• No significant differences among the 3 groups at any time point in weight, length, or head circumference or bone mineral content or density. • Greater (p<0.05) lean mass and reduced fat mass in infants fed formula with LC-PUFA at 12 mo					
• Subset of term infants in the DIAMOND STUDY • Measured growth outcomes from 6 y. • A limitation is the small sample size at 6-year follow-up (n=18-24/group) [186]	• Compared CON formula, children who consumed LC-PUFA supplemented formula had higher length-/stature- and weight-for-age percentiles, but not BMI percentile from birth to 6 y • Maternal smoking predicted lower stature (2-6 y), higher weight-for-length (birth-18 mos) and BMI percentile (2-6 y) independent of LC-PUFA effects. • Gender interacted with the effect of LC-PUFA on stature, and the relationship between smoking and BMI, with a larger effect for boys.					

2′FL, 2′-fucosyllactose; 3′SL, 3′-sialyllactose; 3′FL, 3′-fucosyllactose; 6′SL, 6′-sialyllactose; BF, breastfed; CON, control; GOS, galacto-oligosaccharides; GA, gestational age; HMOs, human milk oligosaccharides; LNnT, lacto-N-neotetraose; LNT, lacto-N-tetraose; LOS, late onset sepsis; LC-PUFA, long-chain PUFA; LRTI, lower respiratory tract infection; MFGM, milk fat globule membrane; NR, not reported; OPN, osteopontin; PC, phosphatidylcholines; PMA, postmenstrual age; RSV, respiratory syncytial virus; SM, sphingomyelins; SF, standard formula; URTI, upper respiratory tract infection.

### Human milk oligosaccharides

Select HMOs tested in RCTs were well-tolerated and supported age-appropriate growth in term infants [136]. Two RCTs investigated either 2′FL alone [137, 138] or 2′FL + LNnT [139,140] (Table 2). Compared with the control formulas, supplemental 2′FL alone modulated circulating and secreted cytokines [138]. Parent-reported frequency of illness and antibiotic use was lower in infants fed formula with 2′FL + LNnT [139]. A shift in the microbiome community types was also observed in infants supplemented with 2′FL + LNnT [140]. A more recent RCT assessed the safety and tolerability of a mixture of 5 HMOs at a total concentration of 5.75 g/L, which confirmed there were no differences in growth or tolerance between infants fed standard formula or formula + 5HMO [141]. While confirming the potential immunomodulatory and bifidogenic actions of these specific HMOs *in vivo,* none of these studies recapitulate the composition or complexity of oligosaccharide structures present in human milk.

Machine learning–based classification tools have been used to investigate mechanistic links between fecal microbiome, metabolome, and gut health markers of infants in the 2′FL + LNnT trial [140] who experienced bronchitis or lower respiratory tract infection (LRTI) than in those who did not [139]. Among the main features that discriminated infants who did not experience any reported bronchitis or LRTI were consumption of HMO-containing formula, higher acetate, fucosylated glycans, and *Bifidobacterium*, as well as lower succinate, butyrate, propionate, and 5-aminovalerate, and *Escherichia*. By univariate analysis, infants experiencing no bronchitis or LRTI showed higher acetate and *B. longum* subsp. *infantis* [124]. Of relevance to the question of how to study the complex biology of human milk, this case study illustrates the potential to apply machine learning to existing data sets to identify predictive biomarkers of infant health outcomes in response to human milk components.

### Lactoferrin

Lactoferrin (LF), a multifunctional, iron-binding protein, comprises approximately 15% to 20% of total protein in human milk [142], and is considered to be an important contributor to human milk bioactivity. Human LF stimulates immune function, cell proliferation, and differentiation and has antibacterial and antiviral activities [[142], [143], [144]]. Bovine milk LF (bLF) shares 67% amino acid sequence homology with human LF, has similar bioactivities [143,145], and has been tested in several RCTs (Table 2) [144,146,147]. Some studies have shown modest improvements in iron status and lower incidence of upper respiratory illness [146,148] and diarrhea [148] with bLF supplementation; however, other studies have shown no effects [149,150]. In very low birth weight infants, early studies showed that giving bLF orally decreased necrotizing enterocolitis (NEC), sepsis and mortality [151, 152]. Recent multicenter RCTs have shown no benefit of oral bLF [153,154]. These divergent findings may be due to different sources of bLF, its heat treatment before administration, or contamination by other proteins [155]. Infants fed formula with both bLF and MFGM showed improved neurodevelopmental outcomes [156], but it is not possible to assess if both components contributed to the improved outcomes as other studies have shown that MFGM alone affects cognitive development. To date no studies have been found that have explored LF from the perspective of the interactions of the triad either in terms of LF composition or impact.

### Milk fat globule membrane

Fats in human milk are packaged within a 3-layered membrane, or MFGM, which contains anti-infective proteins, carbohydrates (sialic acid), and lipids (gangliosides and cholesterol), the latter of which are purported to be involved in neurodevelopment [157]. Readers are referred to the WG 2 report [2] for additional information on the composition MFGM and its potential biological roles in signaling and cell membrane composition.

Formula contains very little or no MFGM as defatted bovine milk is used as the starting material. RCT have investigated supplementation of formula with bovine milk MFGM, which is not identical to human milk MFGM, but contains similar components and at similar levels [158,159] (Table 2). The addition of a bovine milk protein isolate enriched in MFGM at 4% of total protein to an infant formula was associated with significantly lower rates of infection, and in particular otitis media, than in formula alone [159,160]. MFGM-supplemented infants had a lower abundance of oral *Moraxella catarrhalis*, a microorganism commonly linked to otitis media, providing a potential mechanism of action [161]. There were minor effects of MFGM on fecal microbiome composition [63]. At 4 and 6 mo of age, both the fecal [63] and serum metabolomes [64,69] and serum and erythrocyte cell membrane lipidomes [162] differed between infants fed formula with MFGM vs formula alone; however, differences were no longer present at 12 mo. Lastly, at 12 mo of age, infants fed MFGM-containing formula had significantly higher scores in the Bayley III cognitive domain than infants fed formula without MFGM [159], but a follow-up study at 6.5 y of age found no differences in neurodevelopment between children who were fed the MFGM-containing formula compared with unsupplemented formula [163]. Further complicating our understanding of the contribution of MFGM to human milk bioactivity is that the composition of bovine MFGM used in RCTs varies considerably among commercial suppliers [157].

As with the previous components discussed in this section, while there is evidence to suggest a functional impact of the MFGM on infant related outcomes, no studies were found that explored the nature of the triadic relationships on MFGM content or functional impact. Furthermore, a need exists to apply the ecological systems approach advocated throughout the BEGIN WG reports to advance our understanding of the biology of the MFGM not just as a component of human milk, but as a unique biological system in and of itself.

### Osteopontin

OPN is abundant in human milk, but is low in cow’s milk and, consequently, infant formula [164,165]. OPN stimulates cell proliferation and differentiation and exerts immunomodulatory functions by binding to integrin and CD44 receptors on cell membranes [164]. Interestingly, unlike other components reviewed in this section, there is evidence of an important role of the triadic relationship in OPN content and function. For example, factors in the lactating parent such as BMI, birth route, pregnancy weight gain, and energy intake during lactation affect human milk OPN concentrations [166]. Consistent with immunomodulatory activities, negative associations between human milk OPN levels and fever-related infant hospitalizations from 0 to 3 mo of age and have been reported, suggesting an interaction between the infant and OPN content within the human milk matrix [166].

An RCT studied 1- to 6-month-old infants who were fed standard formula, formula with bovine OPN added at half the human milk level (65 mg/L) or formula with the same level as in human milk (130 mg/L) and compared these groups with a BF group [167]. Infants fed formula + OPN had significantly less morbidity (days with fever), lower proinflammatory and higher anti-inflammatory cytokine levels in serum [167], and differences in circulating lymphocyte subsets [168] than infants fed standard formula. A follow-up analysis reported higher human OPN concentrations in plasma of both formula + OPN groups and BF infants than in infants fed standard formula [165]. In addition, plasma bovine OPN concentration was greater in the 130 mg/L OPN group than the 65 mg/L group [165], suggesting that dietary bovine OPN was absorbed and could affect endogenous OPN synthesis and secretion [165]. A finding that OPN and LF form a strong complex that enhances resistance against digestion and several bioactivities [169] should be considered when evaluating the matrix of human milk.

The evidence of the important immunomodulatory role of OPN and the potential impact of the response of both the parent and infant to infection reveals both an intimate relationship between the triad and OPN content, as well as the potential importance of the compound in our response to current and emerging infectious diseases.

### Long-chain polyunsaturated fatty acids

LC-PUFAs are the most studied functional human milk components, with AA, DHA, and EPA being the most commonly studied n-6 LC-PUFAs and n-3 LC-PUFAs, respectively. Over 40 y ago, dietary LC-PUFA intake by the lactating parent was shown to affect human milk. LC-PUFA concentration [170] and human milk LC-PUFA was linked to infant LC-PUFA status [171,172]. At the same time, other investigators showed the importance of DHA for neurodevelopment [173,174] and identified that the period between 24 wk gestation and infancy was critical for brain DHA accumulation [175,176]. At that time, infant formula lacked preformed AAand DHA, precipitating a number of RCTs to study the effects of LC-PUFA supplementation to the lactating parent (see Section 1) or added to formula fed to term or preterm infants. These findings have been reviewed in recent systematic reviews and meta-analyses [[177], [178], [179]] and are briefly summarized in Table 2.

The DHA intake and measurement of neural development (DIAMOND) study is the only dose-response RCT of DHA and AA that investigated the addition of AA (0.64% of total fatty acids) and 4 doses of DHA (0%, 0.32%, 0.64%, and 0.96% DHA) to infant formula, which was fed for the first year of life [180]. In this study, improvements were observed in cortical visual acuity [180] and cognitive outcomes in all DHA groups compared with 0% DHA, some of which persisted through 6-9 y of age (Table 2) [[181], [182], [183]].

However, 2 recent systematic reviews found no benefit of LC-PUFA supplementation on longer-term cognitive outcomes [177,184]. In the first, cognitive performance of term-born children >2.5 y (range 3.3-16 y), assessed by the Wechsler Preschool and Primary Scale of Intelligence-Revised, showed no effect of early LC-PUFA supplementation. Similarly, performance of preterm-born children on the Wechsler Abbreviated Scale of Intelligence was not affected early LC-PUFA supplementation [177]. The same group of investigators compared differences in academic performance of term-born or preterm adolescents who had participated in one of 7 RCTs of formula supplementation as infants, 2 of which were LC-PUFA supplementation studies [184]. At 11 y of age, preterm and term participants randomized to LC-PUFA supplemented formula scored lower in English and mathematics than those not supplemented as infants. These observations highlight not only the need for longer-term follow-up of infant feeding studies but also for addressing heterogeneity in neurocognitive assessment measures.

Differences in growth and body composition in response to LC-PUFA supplementation have been reported in preterm and term infants. Preterm infants fed formula with DHA and AA had increased lean mass and reduced fat mass compared with infants fed formula without LC-PUFA [185]. A follow-up of term infants enrolled in the DIAMOND study [180] found increased length- and weight-for-age, but not higher BMI, through 6 y in those fed LC-PUFA as infants [186]. However, both of these studies were small and a recent Cochrane review of 13 studies that measured physical growth of term infants found no beneficial or harmful effects of supplementation [178].

Lastly, the influence of LC-PUFA supplementation on infant immune development has received considerable attention. In some studies, infants fed formulas with both DHA and AA, exhibited less skin and respiratory allergic diseases in childhood compared with those fed formulas without LC-PUFA [180]. An analysis of infection and allergic outcomes of >8,000 infants enrolled in the ELFE French longitudinal birth cohort reported that consumption of DHA/AA/EPA-enriched formula (especially those with high EPA content) was associated with a lower risk of LRTI and lower use of asthma medications than in infants fed nonenriched formula [187].

The value of systems approach with specific regard to these compounds is that questions remain with regard to need for and value of LC-PUFA at specific times in infant development, e.g., the value of supplementation to preterm vs term infants. A systems approach to the triad would shed light on not only the biology but the metabolism and chronobiology of these compounds to determine the role of parental supply and critical periods of need for the infant.

### Conclusions

**FIGURE 1:**
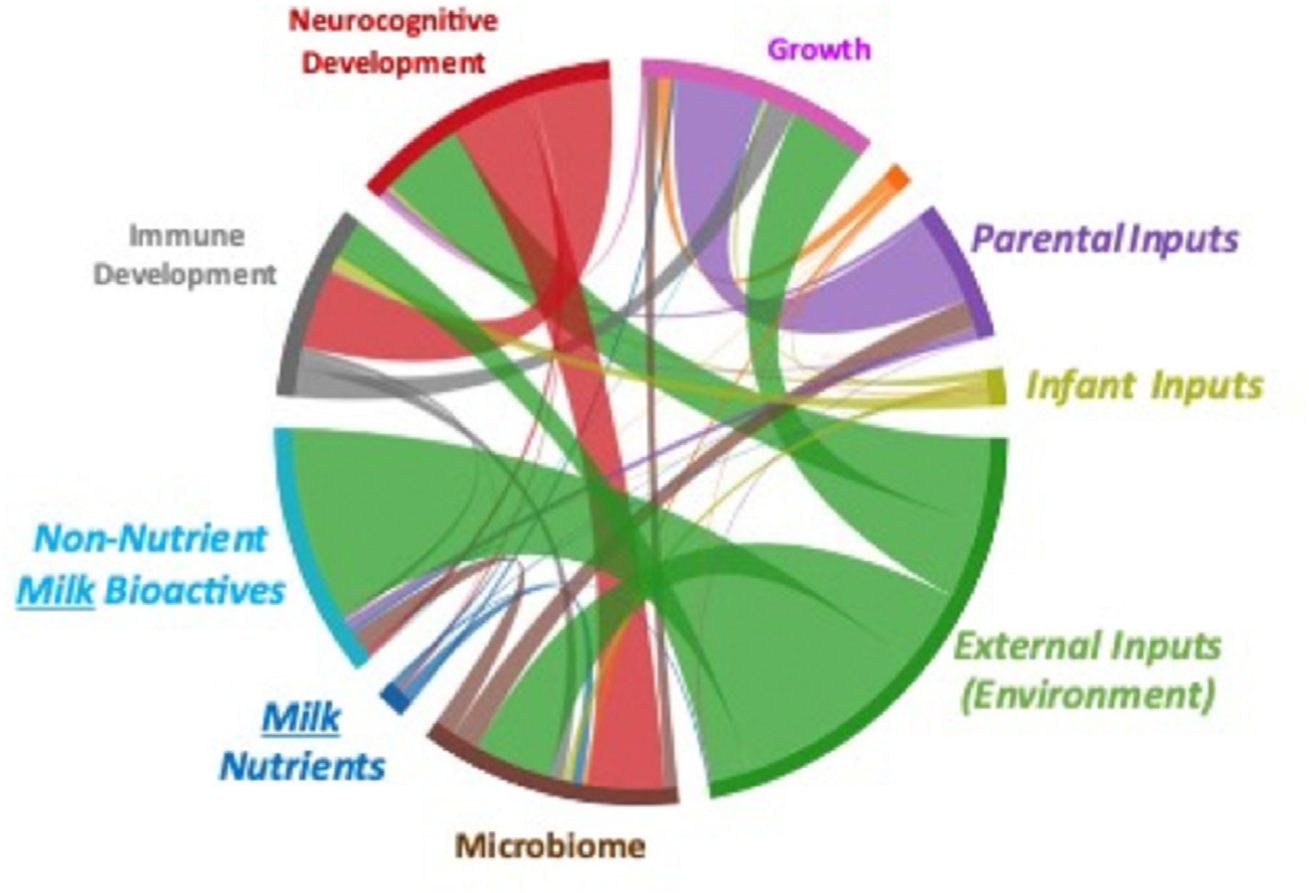
Representative chord diagram illustrating multilevel interactions that require systems biology approaches. Systems biology is an approach in biomedical research to understand the larger picture of interactions within the matrix of human milk, on multiple systems within the infant, which also interact (e.g., the microbiome–gut–brain axis).

This limited review of RCTs has provided evidence that individual human milk components, or their bovine analogs, supplemented to infant formula at levels present in human milk affect specific aspects of infant development. However as illustrated in Table 2 and Figure 1, a single component, such as OPN or MFGM, affects more than one infant outcome (e.g., infection, microbiome, and cognition). These observations reinforce the need to consider interactions among components within the human milk matrix as well as interactions *within the infant* as development of one system in the infant (e.g., microbiome) can impact infant immune, gut, or brain development (Figure 1). For example, HMO may directly affect immune or brain development or may act indirectly via modulation of gut microbial composition or function.

To decipher the complexity of human milk, systems biology approaches, which are based on the understanding that networks that form the whole of living organisms are more than the sum of their parts, will be needed.

## Section 3: Experimental Framework and Analytical Approaches to Study Human Milk as a Complex Biological System in Term Infants

The central premise of the BEGIN project is that human milk is a complex biological system. As discussed throughout the BEGIN reports [[1], [2], [3], [4], [5]], human milk composition consists of many interacting components, is embedded within the lactating parent–human milk–infant triad [188] and is affected by communication between the lactating parent and infant. As highlighted by Smilowitz et al. [2], studying a single element of this triad, or a single component of human milk, ignores their integrated nature and limits our ability to understand the determinants and consequences of human milk composition and function.

**FIGURE 2:**
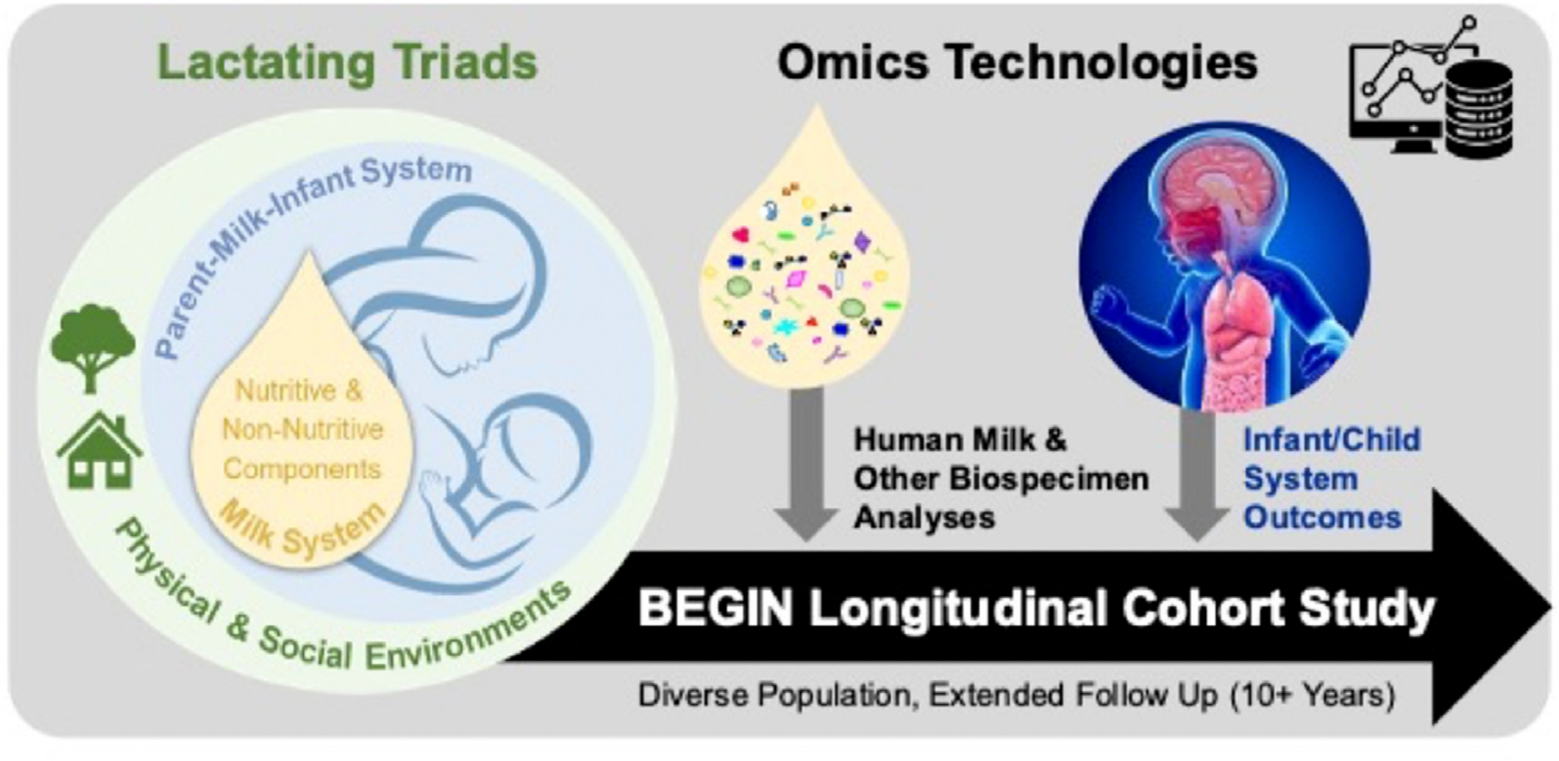
Biological effects of human milk as a system-within-a-system. Human milk is a complex biological system comprising many components within a unique matrix that is imbedded within the lactating parent–human milk–infant triad. Human milk composition is shaped by both external and internal ecologies unique to each lactating parent and infant dyad. Human milk influences the development and maturation of interacting systems within the infant (e.g., immune, microbiome, intestinal, brain), which influence long-term outcomes in the infant.

**FIGURE 3:**
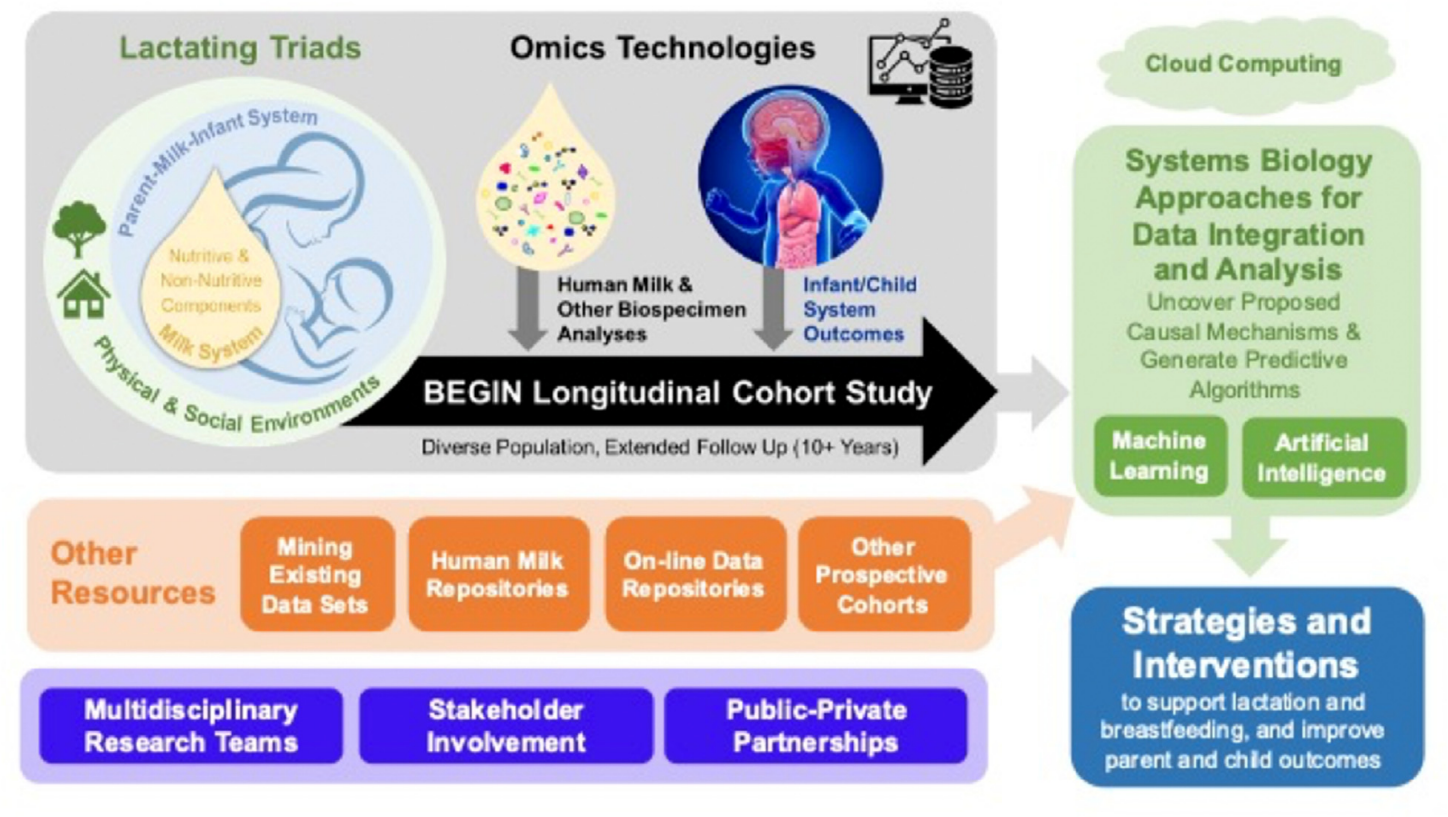
Proposed analytical framework to study human milk as a biological system. To further our understanding, multidisciplinary teams of investigators should undertake a longitudinal investigation of diverse cohort of lactating parent–infant dyads. Frequent biological sample collection coupled with extensive paired metadata and multi-omic analyses with enable deep phenotyping of participants. Application of systems biology provides a unique opportunity for in-depth modeling of the role of human milk in the breastfeeding dyad to develop integrated models that predict and explain the role of human milk on a systems level.

We propose that the biology of breastfeeding and human milk should be studied as a “system-within-a-system” (Figure 2), which has not been fully undertaken to date. The suggested approach will require careful planning from the outset by transdisciplinary teams of researchers to incorporate extensive biological and metadata collection, advanced analytical approaches, and systems biology integration (Figure 3). As will be described below, Foundations (e.g., the Bill and Melinda Gates Foundation) have undertaken multicountry breastfeeding interventions in recent years; however, most have not collected data that will enable full interrogation of the lactating parent–human milk-infant triad. The understanding of the importance and the complexity of the triad by funding agencies will enable resources to be directed to more comprehensive studies in the future. The following sections provide descriptions of key elements of this approach.

### Essential components of the human milk research infrastructure

Studies intended to expand our understanding of the complex biology of human milk, particularly those involving large subject cohorts, will require attention to a number of key elements of study design and relevant technology. One approach would be the establishment of a longitudinal cohort study specifically designed to collect, analyze, and integrate the complexity of human milk as a biological matrix, including an interrogation of the parental and infant factors influencing that complexity, and the short- and long-term implications of human milk on relevant infant and child health outcomes. In addition, a need exists for resources to collect and annotate existing data and biological samples and to generate guidelines for broader data capture for ongoing or future single- or multiinvestigator studies and interventions, which will be essential for advancing the human milk research agenda (Text Box 5).

Text Box 5 **Approaches for gathering existing and future human milk and lactation metadata and biological samples to inform systems biology approaches**

1. Establishment of an *online portal* where researchers can upload demographic and biological data from completed and ongoing studies.
2. Creation of a *biorepository* of human milk samples paired with other biological samples, saliva, stool, urine, etc.
3. Development of a core list of metadata to be collected in studies of human milk, lactation, and breastfeeding practices.
4. Utilize validated surveys/questionnaires when possible or validate newly developed surveys/questionnaires
  a. NIH PhenX Toolkit is a Web-based catalog of recommended protocols [189]
  b. Human milk researchers should contribute protocols to this site
5. Availability of supplemental funding to support collection of additional metadata and biological sample collection on existing or future single-investigator grants.

Alt-text: Text Box 5

In particular, human milk repositories have an important role to play in these efforts. Two examples in North America are the Mommy’s Milk Human Milk Biorepository at the University of California, San Diego [190] and the Manitoba Interdisciplinary Lactation Center [191]. By using standardized protocols for sampling and storage, collecting, and cataloging human milk from large populations with diversity in race and ethnicity, geography, and sociodemographic factors, repositories can serve as a central platform for interdisciplinary human milk research. Individual samples can be aliquoted at the time of processing and then analyzed for multiple components, and/or accessed by multiple investigators with different research questions. Repositories that can link human milk samples to electronic health data for lactating parents and infants are especially powerful, offering low-cost opportunities to perform longitudinal studies on the association of human milk components with lactating parent and child health. When multiple datasets are generated using the same human milk samples, and appropriate data sharing agreements are in place, repositories offer exciting synergistic opportunities to undertake multi-omic analyses on human milk samples from well-characterized triads.

### Specific study considerations

As stated by Steven Covey, “Begin with the End in Mind” [192], which entails creating a clear vision of direction and destination to help in attaining a goal. In this case, the five-stage nonlinear translational framework conceptualized by WG 5 provides an excellent guide to consider how discovery research (T1) could be designed and adapted to inform the other 4 stages: human health implications (T2), clinical and public health implications (T3), implementation (T4), and, ultimately, impact (T5) [4]. The analytical framework to study human milk as a biological system is shown in Figure 3, and the following sections describe specific aspects to be considered in the design of integrated studies of human milk.

#### Consideration of participant selection

Although some global initiatives are underway [191,[193], [194], [195]], the vast majority of human milk research has been conducted in ethnically homogeneous, relatively Western populations. As discussed in more detail by WG 5, with expansion of the human milk and lactation research enterprise to large comprehensive studies to profile human milk, it will be important to ensure that diversity and representation are considered when recruiting research participants. In addition, due to the lack of evidence for long-term outcomes of early life nutrition interventions (e.g., from RCTs of specific bioactive components of human milk as discussed previously) it will be critical that the cohort be followed for a minimum of 10 y. Moreover, longitudinal cohorts will need to be adequately powered to account for attrition and participants should provide consent for future analyses. As single nucleotide polymorphisms in the lactating parent or the infant can influence human milk composition or utilization by the infant, studies should apply Mendelian randomization for common single nucleotide polymorphisms when applicable [196,197]. Lactahub, a partnership project of The Global Health Network at the University of Oxford and the Family Larsson-Rosenquist Foundation, recently published a useful framework to guide ethical breastfeeding research and interventions [198].

As we consider conducting research across the globe, decolonizing research is a complex problem that requires a multipronged approach to affect change. Currently a power imbalance exists between HIC and low- or middle-income income countries (LMIC), which creates and perpetuates imbalance for field sites. Shifting this imbalance must be a priority in the design of future human milk studies. Specifically greater attention, understanding, and funding need to be allocated for utilities such as electricity to facilitate long-term sample storage at LMIC field sites, clinics, and hospitals, as well as infrastructure to support data capture and development of local bioinformatics cores. Intellectual property must be shared with LMIC collaborators in addition to equal acknowledgment for their contributions. Compensation for researchers and staff in LMIC environs is generally budgeted lower than that of HIC counterparts; this perpetuates the misconception that there is a gap in research capacity. Highly skilled researchers are present in LMIC settings, it is a matter of HIC collaborators recognizing that the capacity exists and budgeting appropriately to retain this expertise. Travel for field site visits and conference presentations is often budgeted for HIC research teams; however, LMIC counterparts rarely receive funding in these areas. Trainee exchange programs must be encouraged, as well as financial support for field site investigators and research teams to present findings in their own right rather than the HIC PI presenting on their behalf.

#### The value of collaborative team science

The challenge of studying the complexity of human milk and its ecology will require cross-disciplinary teams of investigators representing multiple disciplines (e.g., cultural and physical anthropology, evolution, immune function, mammary gland biology and lactation physiology, medicine, neurobiology, nutrition, and public health) working together in an integrated approach [199]. While scientific research is increasingly conducted by small teams and larger groups rather than individual investigators, the challenges of managing these collaborations can slow these teams’ progress in achieving their scientific goals. Thus, best practices for enhancing the effectiveness of research teams should be employed [200].

#### Biospecimens and data

In addition to the collection of human milk itself (see [201] for a detailed guide to human milk collection and storage), the study of human milk as a “system-within-a-system” will require additional biological specimens from the lactating parent and infant. Depending on the research question, it may be important to collect blood, urine, infant saliva (a source of human milk microbes and other ‘communication signals’), serum (to monitor nutritional status or hormones), skin, stool, and nasal swabs (to analyze the gut, dermal, and airway microbiomes, respectively). Blood, saliva, urine, feces, and vaginal and skin swabs from the lactating parent may also be relevant.

To assess the impact of lactating parent [1] and infant [3] inputs on human milk composition, it will be necessary to capture information on such factors as dietary intake, feeding practices (frequency, pumping, complementary feeding, etc.), lactating parent and infant health conditions, sociodemographics, as well as the physical and social environment. TABLE 3, TABLE 4 summarize the recommendations for sample and data collection and analyses, respectively, to support systems biology analyses. Although logistically challenging, capturing milk volume (produced by the lactating parent and/or consumed by the infant) provides extremely useful information and would be necessary to determine lactating parent inputs and infant exposure. Researchers are also encouraged to use relatively noninvasive approaches for repeated measures of cognitive development (e.g., magnetic resonance imaging [MRI]) [202,203], body composition (air displacement plethysmography, quantitative nuclear magnetic resonance or dual X-ray absorptiometry) [[204], [205], [206]], or intestinal gene expression using exfoliated epithelial cells [35].

**TABLE 3:** Recommendations for data, testing, and biospecimens to study human milk as a system-within-a-system.

Type of Data	External Ecologies	Internal Ecologies	
		Lactating Parent	Infant or Child
Environmental and biological sample collections	Environmental samples • Water, • Soil • House dust • etc.	• Human milk • Blood • Saliva • Oral and nasal swabs • Skin swabs, including nipple and areola • Hair • Feces • Urine • Vaginal swabs	• Blood • Saliva • Oral and nasal swabs • Skin swabs • Hair • Feces • Urine
Physiological measurements or testing or observations		• Height • Weight • Body composition • Milk production • Brain structure and functional MRI • Endocrine, immune, and intestinal function (see Table 4) • Biopsychosocial interactions between parent and child, particularly during feeding	• Length or height • Weight • Body composition • Milk intake and mode of feeding • Brain structure and functional MRI • Cognitive function (tested) • Endocrine, immune, and intestinal function (see Table 4) • Biopsychosocial interactions between parent and child, particularly during feeding
Medical records, questionnaires, and surveys	• Social determinants of health∗ • Geography • Religion • Culture • Employment type • Sanitation • Stress • Xenobiotic exposure - environmental contaminants, - therapeutic or recreational drugs, - cigarette smoke - other toxins	• Race and ethnicity • Sex • Health history and current status • Medications and supplements • Breastfeeding duration and exclusivity • Sleep patterns • Weight status: prepregnancy, gestational weight gain, recent weight gain or weight loss • Parity • Pregnancy complications • Breast surgery (reduction or augmentation) • Lactation history • Timing of initiation of lactation • Executive function • Physical activity	• Race and ethnicity • Sex • Health history and current status • Medications and supplements • Breastfeeding duration and exclusivity • Sleep patterns • Gestational age • Singleton or multiple birth • Infant formula use and type • Dietary intake (human milk, infant formula, complementary feedings) • Cognitive function (parent reported)

MRI, magnetic resonance imaging.

∗ Social determinants of health are conditions in the places where people live, learn, work, and play that affect a wide range of health and quality-of life-risks and outcomes (https://www.cdc.gov/socialdeterminants/index.htm).

**TABLE 4:** Proposed sample analyses to study human milk as a biological system and interactions within the lactating triad.

Sample Type	Human Milk (Colostrum, Transitional and Mature)	Lactating Parent	Infant or Child
**Genome:** Single nucleotide polymorphisms	Cells from lactating parent	Blood	Blood or saliva
		Saliva	
**Epigenome:** DNA methylation	Cells from lactating parent	Blood	Blood
		Oral or nasal swabs	Oral or nasal swabs
		Saliva	Saliva
**miRNA/exosomes**	Human milk	Blood	Blood
**Nutrients**: Proteins, lipids, HMO, vitamins, minerals, trace elements	Human milk	Blood	Blood
		Urine	Urine
**HMO**	Human milk	Blood	Blood
		Feces	Feces
		Urine	Urine
**Microbiome and metagenome**: Bacteria, fungi, Archaea, viruses	Human milk	Feces	Feces Saliva Swabs: Oral, nasal, skin
		Saliva	
		Swabs: Oral, nasal, vaginal, skin (including nipple and areola)	
**Metabolome and proteome**	Human milk	Blood Feces Urine	Blood Feces Urine
**Lipidome**	Human milk	Blood	Blood
**Immune cells and proteins**	Human milk	Blood	Blood
**Hormones and growth factors**	Human milk	Blood	Blood

HMO, human milk oligosaccharide.

#### Timing and procedure of sample collection

As described by WG 1-3 [[1], [2], [3]], a unique chronobiology exists for human milk composition involving both parental and infant inputs. Temporal changes in human milk composition occur over the course of a feeding, a day and throughout the period of lactation. Timing is thus an important consideration when designing human milk studies. Ideally, the time of day and time postpartum should be standardized across participants, or at least clearly documented so that these parameters can be accounted for during data analysis. Depending on the research question, it may be especially relevant to collect colostrum, transitional, and/or mature milk. Longitudinal sampling can offer important insights about compositional changes over time and can link human milk composition to short- and long-term health outcomes.

In addition, how the human milk sample was collected is a crucial design component as numerous factors can influence accuracy of milk composition. These factors are described in detail elsewhere [201,207,208]. Although many methodological factors (e.g., human milk sampling, handling, and analytics) are known to impact human milk composition, few studies have investigated those as primary outcomes or variables, making it an important area of future research in human milk [207]. Examples of factors to consider are shown in Text Box 6.

Text Box 6 **Factors to consider for standardizing human milk sampling**

• Time of day and if coincident with routine feeding times?
• Time postpartum.
• If or how the breast was cleaned prior to sample collection.
• Was the milk collected by manual expression or breast pump? If a pump, what type?
• Does the milk sample represent an aliquot of a full breast expression or milk sampled early, mid or late during a feeding?
• Were both breasts sampled or, if only one, left or right?
• How was the sample stored after expression, for how long and in what type of container (bag, plastic bottle, glass bottle, etc.), was a preservative added? [207]

Alt-text: Text Box 6

#### Human milk analysis

In addition to the coverage of human milk components offered by WG 2, readers are referred to the recent book summarizing milk sampling and nutrient and macromolecule analysis [209]. It is important to consider at the study design phase which analytes will be analyzed (or might be in the future), as this will inform sample collection, analysis, storage, and requisite resources needed. For example, some components are highly sensitive to light and/or freeze-thaw cycles, whereas others are unaffected [201,207]. It is also important to ensure that assays have been validated for human milk. As for any large study, appropriate controls and standards should be included to address and adjust for potential batch effects.

#### Integration: computational systems biology

The previous sections illustrate the myriad of internal and external ecologies that may affect human milk composition and its impact on the lactating parent–infant dyad. Due to this complexity, outcome-based studies on a population level often only cover very specific aspects of human milk ecology. At the same time, recent advances in high-throughput technologies have provided access to multidomain and multi-omic data, enabling unprecedented insights into complex biological systems from multiple viewpoints such as the microbiome, proteome or metabolome, as well as a wide range of other omics [210]. Such technologies will require systems biology approaches to study human milk as a complex biological system [6], unraveling the underlying processes that influence human milk composition, and its effects on infant development and clinical/functional outcomes in both infant and lactating parent.

In contrast to population level approaches, multi-omic studies typically exhibit different data characteristics and challenges (Text Box 7). High-through put multi-omics data poses unique challenges and requires sophisticated computational tools to analyze. For this, a wide variety of methods for multi-omics integration are currently being developed at the intersection of bioinformatics and machine learning [[211], [212], [213], [214]]. This includes methods based on specific fields like Bayesian modeling [215,216], network analysis [213,214] as well as deep learning [217,218], and considerations on sample size requirements and estimation [219,220]. In addition, there are community-driven efforts to maintain an overview of relevant work and software packages [221].

Text Box 7 **Analytic challenges of multi-omic studies**

• Large numbers of measurements across several omics technologies per sample, while the number of samples is small (commonly machine learning models are trained and optimized on datasets with many more samples than measurements)
• Different omics techniques are highly heterogeneous in individual characteristics and number of features (e.g., sparse microbiome data vs. targeted proteomics assays). In particular, sparse data with many undefined values require different processing and model imperatives than data with dense input, and
• Omics data of high dimensionality, but with low information content, may preclude information from smaller, more dense omics to be included in a model. Stack generalization has previously been utilized successfully to address this challenge
• Omics data from various study sites may be impacted by a combination of genetic, environmental, and technical factors. Careful use of machine learning algorithms can enable the development of generalizable models despite these variations

Alt-text: Text Box 7

While integrating multiple omic datasets is challenging, it has been demonstrated to improve the capacities of models for biological processes in the context of the lactating parent–infant dyad, particularly as they relate to pregnancy outcomes. For example, several studies illustrate that combining the information from a wide variety of omics—including transcriptome, or proteome, and more—can be used to model gestational age [222], as well as improve the performance of predictive models for adverse pregnancy outcomes like preterm birth [223], preeclampsia [224], or gestational diabetes [225]. Additionally, these models help to elucidate complex biological systems, as exemplified by a recent study mapping the underlying processes of onset of labor [226].

Applying and adjusting these methods to studying human milk holds great potential to uncover the complex processes involved in the triad from a systems biology viewpoint. A particular challenge in this context is to integrate population level studies with multi-omics approaches due to the demanding collection process of synchronized omic measurements as well as the technical aspects of integrating omics with nonomics records, such as clinical meta data [227,228]. However, multimodal models have been previously applied in biological data integration [229] and are particularly relevant for multi-omics analysis and integration [147,214] with the potential to combine modalities across regular tabular data, time series, images, as well as text into a joint holistic model for a multitude of predictive settings [230,231].

Human milk and feeding within the breastfeeding dyad influence infant development as well as various clinical/functional outcomes. These effects are highly interrelated and may point toward intertwined underlying processes. This interrelatedness can be studied and can be exploited by using novel concepts from machine learning in the field of multitask learning [232]. Multitask learning takes advantage of information contained in related outcomes to make models more robust by preferring solutions that share common (e.g., biological) structures across these outcomes [233]. Here, deep learning has recently contributed significantly to advancing the field of multitask learning [234,235].

In summary, recent advances in machine learning, including multimodal learning, multi-view representation learning [230,236] and multitask learning [233,235] provide a unique opportunity for in-depth modeling of the role of human milk in the breastfeeding dyad. These areas of research aim to combine datasets from various modalities and across different tasks (e.g., prediction of outcomes) to develop integrated models that predict and explain the role of human milk on a systems level and can apply techniques currently used in other areas, such as microbial community ecology [237]. The combination of multimodal learning with multitask approaches [238] allows for condensed representations of the inputs and the modeled phenotypes (outcomes), which may lead to a novel holistic understanding of the underlying processes associated with human milk and its influence on infant development as well as clinical/functional outcomes.

### Case study 1—interactions of human milk components and cow milk allergy

Recently, hierarchial cluster analyses have been used to assess correlations between immune factors in human milk showing that those factors (IL-6, IL1β, IL-10, thymic stromal lymphopoietin, TGF-β1) that were correlated with infant cow’s milk allergy as an outcome were actually independent of maternal atopy [58]. A follow-up study in a farming lifestyle cohort utilized systems level network analysis to identify communities of multiple human milk factors [239]. Path-based analysis of HMO showed lower activity in the path involving lactoneohexaose in the farming lifestyle mothers as well as higher levels of LNnT and 2 long-chain fatty acids (stearic acid and tricosanoic acid) compared with urban lifestyle mothers. Human milk from both groups formed 5 different clusters, e.g., butyrate production was associated with *Prevotellaceae, Veillonellaceae, and Micrococcaceae* cluster. Development of atopic disease in early childhood was more common in urban lifestyle and associated with lower levels of total IgA and IgA_2_ to dust mite, as well as of thymic stromal lymphopoietin. Thus, external ecologies (traditional, agrarian lifestyle, and antibiotic use) were shown to be strong regulators of immune and metabolic factors derived from the lactating parent, which may have downstream implications for postnatal developmental programming of infant’s gut microbiome and immune system [239].

### Case study 2—lactating parent–human milk-infant interacting microbiomes

In the BEGIN Project Executive Summary [5], lactating parent–human milk-infant interacting microbiomes were highlighted as a Case Study for the need to apply an ecological systems biology approach. A consortium of microbes, viruses, archaea and fungi and protozoa are present in human milk and are proposed to play a role in early infant colonization [240]. The large number of host, microbial, and medical factors that can influence the human milk microbiota complicate our understanding of the factors driving heterogeneity in the human milk microbiome [195]. Furthermore, the relationships between the human milk microbiome and infant microbial colonization are poorly understood, with 2 studies reporting that 25% [241] or >70% [242] of the infant fecal genera originate from human milk; however, neither study reported strain level analysis. Expression of human milk via pumping also affects the human milk microbiome [243] and is often not reported in studies of the human milk microbiome.

In the CHILD cohort, co-occurrence of specific bacteria in human milk and infant feces (including *Streptococcus* spp. and *Veillonella dispar*) were observed within mother–infant dyads, which was reduced when infants received pumped human milk in a bottle [244]. The commonly shared human milk bacteria were associated with the 3-month-old infant gut microbiota, explaining 1.0% of the variation (R^2^_adj_). While this is a low percentage, it was greater than that explained by breastfeeding exclusivity (0.76%), birth mode (0.87%), intrapartum antibiotics (0.72%), or older siblings (0.58%) [244]. Multivariate analyses were applied to investigate the contribution of bacterial communities across the lactating parent–infant dyad (maternal and infant saliva, human milk, maternal and infant feces) on human milk and infant fecal bacterial composition [85]. As might be expected, the human milk microbiome was more similar to the infant oral microbiome than the infant fecal microbiome. Canonical correlation analysis suggested strong associations between the human milk genera and all other sample types, although sources of >87% of the infant fecal genera were unknown based on SourceTracker2. However, this might be a limitation of the program and the depth of sequencing because species or strain level resolution was not reported [85]. Furthermore, only 52% of infants were exclusively BF at 3 mo of age and use of breast pumps was not reported [85]. Another study also utilizing SourceTracker identified infant gut microbiome composition more similar to sibling stool samples than human milk [245]. Taken together, these data demonstrate potential relationships between human milk and infant fecal microbiota; however, more comprehensive data collection and human milk sample analyses are required to delineate other ecological factors within the lactating parent–human milk-infant triad that are accounting for infant microbiome composition.

### Overall Conclusions

While there are notable challenges in the study of human milk as a complex biological system, emerging scientific and analytical advances are uncovering unprecedented opportunities to study the biology of human milk and uncover the internal and external ecologies that drive outcomes observed in the breastfeeding triad over time and across a myriad of conditions. Considerations for the design of human studies of these complex relationships, including the integration of new approaches in multi-omic data collection, computational systems biology and approaches to the use of large complex datasets, will be important to advance the field of human milk and lactation research. Unprecedented opportunities await the field to uncover new mechanistic interactions between the triad of the human milk biological system. The challenges remain great, but the return will be greater.

## Funding

There was no committed funding for the writing of this manuscript for any author. However, during the period of manuscript preparation authors were funded from the following sources: AA (USDA-ARS Project Plan 6026-51000-010-05S, NIH R01 DK107516, NIH R01 ES032176, NIH U01DA055352, NIH R01 HD099099-01); NA (NIH R35 GM138353, Burroughs Wellcome Fund, the Bill and Melinda Gates Foundation, March of Dimes); MBA (Tier 2 Canada Research Chair in the Developmental Origins of Chronic Disease at the University of Manitoba and is a Fellow in the Canadian Institutes for Advanced Research (CIFAR) Humans and the Microbiome Program. Research funding from the Canadian Institutes of Health Research, Research Manitoba, the Canada Foundation for Innovation, the Bill and Melinda Gates Foundation, the Manitoba Children's Hospital Foundation, Prolacta Biosciences, Mitacs, CIFAR, the GarfieldWeston Foundation, Health Data Research UK, and Canadian COVID Immunity Task Force); MB (NIH R35 GM138353, Burroughs Wellcome Fund, the Bill and Melinda Gates Foundation, March of Dimes); SEC (NIH R01 HD083292, NIH R01 HD086001-A1, NIH R01 DK118220-01, DSM, Mead Johnson Nutrition, Danone); SMD (NIH R01 DK107561, NIH R01 HL153306, NIH R01 GM127347, USDA AFRI 2016-08909, ByHeart, Gerber Foundation, International Flavors and Fragrances, Kyowa Hakka Bio, National Dairy Council); KJ (NIH U01 AI131344, NIH U01 AI131344-04S1, NIH R21 AI163571, R03 AI151965, Aimmune, Janssen R&D); WL (NIH U01 MH110274, NIH R34 DA050262, Nestec S.A.); BL (ByHeart); CS (NIH R21 AG071156; USDA-NIFA 2021-67017-35783; USDA-NIFA Hatch project 1021411; Arla Foods Ingredients; Kinsella Endowed Chair in Food, Nutrition, and Health).

The BEGIN Project was initiated by the Pediatric Growth and Nutrition Branch of the *Eunice Kennedy Shriver* National Institute of Child Health and Human Development (NICHD) of the NIH in partnership with the Bill & Melinda Gates Foundation and the Academy of Nutrition and Dietetics. The publication of this supplement was made possible by the NICHD, and support for assistance (by BioCentric, Inc.) with editing, proofing, and submitting the manuscript was also provided by the NICHD.

## Acknowledgements

The authors acknowledge contributions by Natalie Rodriguez MBA, ACC, CCIP, Director of Operations, Diversity & Inclusion - THRiVE Discovery Lab at Children’s Hospital Research Institute of Manitoba for her contributions on how diversity, equity and inclusion can be applied to human milk research. We also acknowledge the role of BioCentric, Inc. (Collingswood, New Jersey) and its staff (particularly Kevin Jarvis, PharmD; and Andrea Tucker, MA, ELS) in editing and formatting the manuscript in accordance with the journal style, and assisting with the manuscript submission process.

## Author contribution

The contributions of the authors were as follows—DJR conceptualized the project; all authors contributed to writing the manuscript: Introduction led by DJR and SMD, Section 1 led by AA, SEC, SMD, KMJ, WL, and CMS; Section 2 led by SEC, SMD, BL, and CMS; Section 3 led by NA, MBA, MB and SMD. SMD, DJR and AS prepared the final draft of the manuscript, which was read and approved by all authors.

## Conflicts of interest

All authors have declared no conflicts of interest related to existing financial arrangements with any company or organization sponsoring this manuscript. (AS is an employee of the Academy of Nutrition and Dietetics).

## References

[1] Neville M.C., Demerath E.W., Hahn-Holbrook J. (2023). Parental factors that impact the ecology of human mammary development, milk secretion, and milk composition—a report from “Breastmilk Ecology: Genesis of Infant Nutrition (BEGIN)” Working Group 1. Am. J. Clin. Nutr..

[2] Smilowitz J.T., Allen L.H., Dallas D.C. (2023). Ecologies, synergies, and biological systems shaping human milk composition—a report from “Breastmilk Ecology: Genesis of Infant Nutrition (BEGIN)” Working Group 2. Am. J. Clin. Nutr..

[3] Krebs N.F., Belfort M.B., Meier P.P. (2023). Infant factors that impact the ecology of human milk secretion and composition—a report from “Breastmilk Ecology: Genesis of Infant Nutrition (BEGIN)” Working Group 3. Am. J. Clin. Nutr..

[4] Nommsen-Rivers L., Black M.M., Christian P. (2023). An equitable, community-engaged translational framework for science in human lactation and infant feeding—a report from “Breastmilk Ecology: Genesis of Infant Nutrition (BEGIN)” Working Group 5. Am. J. Clin. Nutr..

[5] Raiten D.J., Steiber A.L., Papoutsakis C. (2023). The “Breastmilk Ecology: Genesis of Infant Nutrition (BEGIN)” Project: executive summary. Am. J. Clin. Nutr..

[6] Christian P., Smith E.R., Lee S.E., Vargas A.J., Bremer A.A., Raiten D.J. (2021). The need to study human milk as a biological system. Am. J. Clin. Nutr..

[7] Hou L., Li X., Yan P., Li Y., Wu Y., Yang Q. (2021). Impact of the duration of breastfeeding on the intelligence of children: a systematic review with network meta-analysis. Breastfeed. Med..

[8] Bernard J.Y., Armand M., Peyre H., Garcia C., Forhan A., De Agostini M. (2017). Breastfeeding, polyunsaturated fatty acid levels in colostrum and child intelligence quotient at age 5-6 years. J. Pediatr..

[9] Gustafsson P.A., Duchén K., Birberg U., Karlsson T. (2004). Breastfeeding, very long polyunsaturated fatty acids (PUFA) and IQ at 6 1/2 years of age. Acta Paediatr.

[10] Dalmeijer G.W., Wijga A.H., Gehring U., Renders C.M., Koppelman G.H., Smit H.A. (2016). Fatty acid composition in breastfeeding and school performance in children aged 12 years. Eur. J. Nutr..

[11] de la Garza Puentes A., Martí Alemany A.M., Chisaguano A.M., Montes Goyanes R., Castellote A.I., Torres-Espínola F.J. (2019). The effect of maternal obesity on breast milk fatty acids and its association with infant growth and cognition-the PREOBE follow-UP. Nutrients.

[12] Cheatham C.L., Sheppard K.W. (2015). Synergistic effects of human milk nutrients in the support of infant recognition memory: an observational study. Nutrients.

[13] Delgado-Noguera M.F., Calvache J.A., Bonfill Cosp X., Kotanidou E.P., Galli-Tsinopoulou A. (2015). Supplementation with long chain polyunsaturated fatty acids (LCPUFA) to breastfeeding mothers for improving child growth and development. Cochrane Database Syst. Rev..

[14] Lehner A., Staub K., Aldakak L., Eppenberger P., Rühli F., Martin R.D. (2021). Impact of omega-3 fatty acid DHA and EPA supplementation in pregnant or breast-feeding women on cognitive performance of children: systematic review and meta-analysis. Nutr. Rev..

[15] Deoni S., Dean D., Joelson S., O'Regan J., Schneider N. (2018). Early nutrition influences developmental myelination and cognition in infants and young children. Neuroimage.

[16] Wang B., McVeagh P., Petocz P., Brand-Miller J. (2003). Brain ganglioside and glycoprotein sialic acid in breastfed compared with formula-fed infants. Am. J. Clin. Nutr..

[17] Nevins J.E.H., Donovan S.M., Snetselaar L., Dewey K.G., Novotny R., Stang J. (2021). Omega-3 fatty acid dietary supplements consumed during pregnancy and lactation and child neurodevelopment: A systematic review. J. Nutr..

[18] Horta B.L., de Lima N.P. (2019). Breastfeeding and type 2 diabetes: systematic review and meta-analysis. Curr. Diab. Rep..

[19] Patelarou E., Girvalaki C., Brokalaki H., Patelarou A., Androulaki Z., Vardavas C. (2012). Current evidence on the associations of breastfeeding, infant formula, and cow's milk introduction with type 1 diabetes mellitus: a systematic review. Nutr. Rev..

[20] Güngör D., Nadaud P., LaPergola C.C., Dreibelbis C., Wong Y.P., Terry N. (2019). Infant milk-feeding practices and diabetes outcomes in offspring: a systematic review. Am. J. Clin. Nutr..

[21] Cardwell C.R., Stene L.C., Ludvigsson J., Rosenbauer J., Cinek O., Svensson J. (2012). Breast-feeding and childhood-onset type 1 diabetes: a pooled analysis of individual participant data from 43 observational studies. Diabetes Care.

[22] Owen C.G., Martin R.M., Whincup P.H., Smith G.D., Cook D.G. (2006). Does breastfeeding influence risk of type 2 diabetes in later life? A quantitative analysis of published evidence. Am. J. Clin. Nutr..

[23] Bjerregaard L.G., Pedersen D.C., Mortensen E.L., Sørensen T.I.A., Baker J.L. (2019). Breastfeeding duration in infancy and adult risks of type 2 diabetes in a high-income country. Matern. Child Nutr..

[24] Catassi C., Bonucci A., Coppa G.V., Carlucci A., Giorgi P.L. (1995). Intestinal permeability changes during the first month: effect of natural versus artificial feeding. J. Pediatr. Gastroenterol. Nutr..

[25] Newburg D.S. (2005). Innate immunity and human milk. J. Nutr..

[26] Vatanen T., Franzosa E.A., Schwager R., Tripathi S., Arthur T.D., Vehik K. (2018). The human gut microbiome in early-onset type 1 diabetes from the TEDDY study. Nature.

[27] Gao C., Miller J., Collins C.T., Rumbold A.R. (2020). Comparison of different protein concentrations of human milk fortifier for promoting growth and neurological development in preterm infants. Cochrane Database Syst. Rev..

[28] Singh A., Enjapoori A.K., Gibert Y., Dwyer K.M. (2020). The protective effects of human milk-derived peptides on the pancreatic islet biology. Biol. Open.

[29] Xiao L., Van’t Land B., Engen P.A., Naqib A., Green S.J., Nato A. (2018). Human milk oligosaccharides protect against the development of autoimmune diabetes in NOD-mice. Sci. Rep..

[30] Smyth D.J., Cooper J.D., Howson J.M., Clarke P., Downes K., Mistry T. (2011). FUT2 nonsecretor status links type 1 diabetes susceptibility and resistance to infection. Diabetes.

[31] Mirza A.H., Kaur S., Nielsen L.B., Størling J., Yarani R., Roursgaard M. (2019). Breast milk-derived extracellular vesicles enriched in exosomes from mothers with type 1 diabetes contain aberrant levels of microRNAs. Front. Immunol..

[32] El-Amir M.I., El-Feky M.A., ELAbd A., El-Melegy T.T., J I. (2019). HLA-B∗08 carry a risk for type 1 diabetes among cow’s milk exposed Egyptian infants and unmarked linkage disequilibrium with DR3-DQA1∗05-DQB1∗02 haplotype. Egypt. J. Immunol..

[33] Thompson F.M., Catto-Smith A.G., Moore D., Davidson G., Cummins A.G. (1998). Epithelial growth of the small intestine in human infants. J. Pediatr. Gastroenterol. Nutr..

[34] Weaver L.T., Laker M.F., Nelson R., Lucas A. (1987). Milk feeding and changes in intestinal permeability and morphology in the newborn. J. Pediatr. Gastroenterol. Nutr..

[35] Chapkin R.S., Zhao C., Ivanov I., Davidson L.A., Goldsby J.S., Lupton J.R. (2010). Noninvasive stool-based detection of infant gastrointestinal development using gene expression profiles from exfoliated epithelial cells. Am. J. Physiol. Gastrointest. Liver Physiol..

[36] Schwartz S., Friedberg I., Ivanov I.V., Davidson L.A., Goldsby J.S., Dahl D.B. (2012). A metagenomic study of diet-dependent interaction between gut microbiota and host in infants reveals differences in immune response. Genome Biol.

[37] Hawkes J.S., Neumann M.A., Gibson R.A. (1999). The effect of breast feeding on lymphocyte subpopulations in healthy term infants at 6 months of age. Pediatr. Res..

[38] Carver J.D., Pimentel B., Wiener D.A., Lowell N.E., Barness L.A. (1991). Infant feeding effects on flow cytometric analysis of blood. J. Clin. Lab. Anal..

[39] Jeppesen D.L., Hasselbalch H., Lisse I.M., Ersbøll A.K., Engelmann M.D. (2004). T-lymphocyte subsets, thymic size and breastfeeding in infancy. Pediatr. Allergy Immunol..

[40] Wood H., Acharjee A., Pearce H., Quraishi M.N., Powell R., Rossiter A. (2021). Breastfeeding promotes early neonatal regulatory T-cell expansion and immune tolerance of non-inherited maternal antigens. Allergy.

[41] Jansen M.A., van den Heuvel D., van Zelm M.C., Jaddoe V.W., Hofman A., de Jongste J.C. (2015). Decreased memory B cells and increased CD8 memory T cells in blood of breastfed children: the generation R study. PLoS One.

[42] Kainonen E., Rautava S., Isolauri E. (2013). Immunological programming by breast milk creates an anti-inflammatory cytokine milieu in breast-fed infants compared to formula-fed infants. Br. J. Nutr..

[43] Roberts S.A., Freed D.L. (1977). Neonatal IgA secretion enhanced by breast feeding. Lancet.

[44] Taylor C.E., Toms G.L. (1984). Immunoglobulin concentrations in nasopharyngeal secretions. Arch. Dis. Child..

[45] Piirainen L., Pesola J., Pesola I., Komulainen J., Vaarala O. (2009). Breastfeeding stimulates total and cow’s milk-specific salivary IgA in infants. Pediatr. Allergy Immunol..

[46] Hibel L.C., Schiltz H. (2016). Maternal and infant secretory immunoglobulin A across the peripartum period. J. Hum. Lact..

[47] Hasselbalch H., Engelmann M.D., Ersboll A.K., Jeppesen D.L., Fleischer-Michaelsen K. (1999). Breast-feeding influences thymic size in late infancy. Eur. J. Pediatr..

[48] Ogawa J., Sasahara A., Yoshida T., Sira M.M., Futatani T., Kanegane H. (2004). Role of transforming growth factor-beta in breast milk for initiation of IgA production in newborn infants. Early Hum. Dev..

[49] Sjögren Y.M., Tomicic S., Lundberg A., Böttcher M.F., Björkstén B., Sverremark-Ekström E. (2009). Influence of early gut microbiota on the maturation of childhood mucosal and systemic immune responses. Clin. Exp. Allergy.

[50] Ngom P.T., Collinson A.C., Pido-Lopez J., Henson S.M., Prentice A.M., Aspinall R. (2004). Improved thymic function in exclusively breastfed infants is associated with higher interleukin 7 concentrations in their mothers’ breast milk. Am. J. Clin. Nutr..

[51] Hossny E.M., El-Ghoneimy D.H., El-Owaidy R.H., Mansour M.G., Hamza M.T., El-Said A.F. (2020). Breast milk interleukin-7 and thymic gland development in infancy. Eur. J. Nutr..

[52] van Odijk J., Kull I., Borres M.P., Brandtzaeg P., Edberg U., Hanson L.A. (2003). Breastfeeding and allergic disease: a multidisciplinary review of the literature (1966-2001) on the mode of early feeding in infancy and its impact on later atopic manifestations. Allergy.

[53] Lodge C.J., Tan D.J., Lau M.X., Dai X., Tham R., Lowe A.J. (2015). Breastfeeding and asthma and allergies: a systematic review and meta-analysis. Acta Paediatr.

[54] Lionetti E., Castellaneta S., Francavilla R., Pulvirenti A., Tonutti E., Amarri S. (2014). Introduction of gluten, HLA status, and the risk of celiac disease in children. N. Engl. J. Med..

[55] Järvinen K.M., Westfall J.E., Seppo M.S., James A.K., Tsuang A.J., Feustel P.J. (2014). Role of maternal elimination diets and human milk IgA in the development of cow's milk allergy in the infants. Clin. Exp. Allergy.

[56] Järvinen K.M., Laine S.T., Järvenpää A.L., Suomalainen H.K. (2000). Does low IgA in human milk predispose the infant to development of cow's milk allergy?. Pediatr. Res..

[57] Khaleva E., Gridneva Z., Geddes D.T., Oddy W.H., Colicino S., Blyuss O. (2019). Transforming growth factor beta in human milk and allergic outcomes in children: A systematic review. Clin. Exp. Allergy.

[58] Järvinen K.M., Suárez-Fariñas M., Savilahti E., Sampson H.A., Berin M.C. (2015). Immune factors in breast milk related to infant milk allergy are independent of maternal atopy. J. Allergy Clin. Immunol..

[59] Dawod B., Haidl I.D., Azad M.B., Marshall J.S. (2020). Toll-like receptor 2 impacts the development of oral tolerance in mouse pups via a milk-dependent mechanism. J. Allergy Clin. Immunol..

[60] Seppo A.E., Autran C.A., Bode L., Järvinen K.M. (2017). Human milk oligosaccharides and development of cow's milk allergy in infants. J. Allergy Clin. Immunol..

[61] Miliku K., Robertson B., Sharma A.K., Subbarao P., Becker A.B., Mandhane P.J. (2018). Human milk oligosaccharide profiles and food sensitization among infants in the CHILD study. Allergy.

[62] Lodge C.J., Lowe A.J., Milanzi E., Bowatte G., Abramson M.J., Tsimiklis H. (2021). Human milk oligosaccharide profiles and allergic disease up to 18 years. J. Allergy Clin. Immunol..

[63] He X., Parenti M., Grip T., Lönnerdal B., Timby N., Domellöf M. (2019). Fecal microbiome and metabolome of infants fed bovine MFGM supplemented formula or standard formula with breast-fed infants as reference: a randomized controlled trial. Sci. Rep..

[64] He X., Parenti M., Grip T., Domellöf M., Lönnerdal B., Hernell O. (2019). Metabolic phenotype of breast-fed infants, and infants fed standard formula or bovine MFGM supplemented formula: a randomized controlled trial. Sci. Rep..

[65] Fleddermann M., Demmelmair H., Grote V., Nikolic T., Trisic B., Koletzko B. (2014). Infant formula composition affects energetic efficiency for growth: the BeMIM study, a randomized controlled trial. Clin. Nutr..

[66] X. He, C.M. Slupsky, J.W. Dekker, N.W. Haggarty, B. Lönnerdal, Integrated role of Bifidobacterium animalis subsp. lactis supplementation in gut microbiota, immunity, and metabolism of infant rhesus monkeys. mSystems 1(6), 10.1128/mSystems.00128-16. PMC5128019 27921083

[67] He X., Sotelo-Orozco J., Rudolph C., Lönnerdal B., Slupsky C.M. (2019). The role of protein and free amino acids on intake, metabolism, and gut microbiome: A comparison between breast-fed and formula-fed rhesus monkey infants. Front. Pediatr..

[68] Kirchberg F.F., Harder U., Weber M., Grote V., Demmelmair H., Peissner W. (2015). Dietary protein intake affects amino acid and acylcarnitine metabolism in infants aged 6 months. J. Clin. Endocrinol. Metab..

[69] Lee H., Slupsky C.M., Heckmann A.B., Christensen B., Peng Y., Li X. (2021). Milk fat globule membrane as a modulator of infant metabolism and gut microbiota: A formula supplement narrowing the metabolic differences between breastfed and formula-fed infants. Mol. Nutr. Food Res..

[70] Martin F.P., Moco S., Montoliu I., Collino S., Da Silva L., Rezzi S. (2014). Impact of breast-feeding and high- and low-protein formula on the metabolism and growth of infants from overweight and obese mothers. Pediatr. Res..

[71] O'Sullivan A., He X., McNiven E.M., Haggarty N.W., Lönnerdal B., Slupsky C.M. (2013). Early diet impacts infant rhesus gut microbiome, immunity, and metabolism. J. Proteome Res..

[72] Slupsky C.M., He X., Hernell O., Andersson Y., Rudolph C., Lönnerdal B. (2017). Postprandial metabolic response of breast-fed infants and infants fed lactose-free vs regular infant formula: A randomized controlled trial. Sci. Rep..

[73] Brink L.R., Mercer K.E., Piccolo B.D., Chintapalli S.V., Elolimy A., Bowlin A.K. (2020). Neonatal diet alters fecal microbiota and metabolome profiles at different ages in infants fed breast milk or formula. Am. J. Clin. Nutr..

[74] Forbes J.D., Azad M.B., Vehling L., Tun H.M., Konya T.B., Guttman D.S. (2018). Association of exposure to formula in the Hospital and subsequent infant feeding practices with gut microbiota and risk of overweight in the first year of life. JAMA Pediatr.

[75] Stewart C.J., Ajami N.J., O’Brien J.L., Hutchinson D.S., Smith D.P., Wong M.C. (2018). Temporal development of the gut microbiome in early childhood from the TEDDY study. Nature.

[76] Karav S., Le Parc A., Leite Nobrega de Moura Bell J.M., Frese S.A., Kirmiz N., Block D.E. (2016). Oligosaccharides released from milk glycoproteins are selective growth substrates for infant-associated bifidobacteria. Appl. Environ. Microbiol..

[77] R.L. Beverly, P. Woonnimani, B.P. Scottoline, J. Lueangsakulthai, D.C. Dallas, Peptides from the intestinal tract of breast milk-fed infants have antimicrobial and bifidogenic activity. Int. J. Mol. Sci. 22(5), 10.3390/ijms22052377. PMC7956819 33673498

[78] C. Sánchez, C. Fente, P. Regal, A. Lamas, M.P. Lorenzo, Human milk oligosaccharides (HMOs) and infant microbiota: A scoping review. Foods 10(6), 10.3390/foods10061429. PMC8234547 34203072

[79] M. Sakanaka, A. Gotoh, K. Yoshida, T. Odamaki, H. Koguchi, J.Z. Xiao, et al., Varied pathways of infant gut-associated Bifidobacterium to assimilate human milk oligosaccharides: prevalence of the gene set and its correlation with bifidobacteria-rich microbiota formation. Nutrients 12(1), 10.3390/nu12010071. PMC7019425 31888048

[80] Lewis Z.T., Totten S.M., Smilowitz J.T., Popovic M., Parker E., Lemay D.G. (2015). Maternal fucosyltransferase 2 status affects the gut bifidobacterial communities of breastfed infants. Microbiome.

[81] Lewis Z.T., Sidamonidze K., Tsaturyan V., Tsereteli D., Khachidze N., Pepoyan A. (2017). The fecal microbial community of breast-fed infants from armenia and georgia. Scientific Rep.

[82] Liu F., Yan J., Wang X., Wang C., Chen L., Li Y. (2021). Maternal fucosyltransferase 2 status associates with the profiles of human milk oligosaccharides and the fecal microbiota composition of breastfed infants. J. Agric. Food Chem..

[83] Luna E., Parkar S.G., Kirmiz N., Hartel S., Hearn E., Hossine M. (2022). Utilization efficiency of human milk oligosaccharides by human-associated akkermansia is strain dependent. Appl. Environ. Microbiol..

[84] Korpela K., Salonen A., Hickman B., Kunz C., Sprenger N., Kukkonen K. (2018). Fucosylated oligosaccharides in mother's milk alleviate the effects of caesarean birth on infant gut microbiota. Sci. Rep..

[85] Williams J.E., Carrothers J.M., Lackey K.A., Beatty N.F., Brooker S.L., Peterson H.K. (2019). Strong multivariate relations exist among milk, oral, and fecal microbiomes in mother-infant dyads during the first six months postpartum. J. Nutr..

[86] C.A. Moubareck, Human milk microbiota and oligosaccharides: A glimpse into benefits, diversity, and correlations. Nutrients 13(4), 10.3390/nu13041123. PMC8067037 33805503

[87] R.M. Pace, J.E. Williams, B. Robertson, K.A. Lackey, C.L. Meehan, W.J. Price, et al, Variation in human milk composition is related to differences in milk and infant fecal microbial communities. Microorganisms 9(6), 10.3390/microorganisms9061153. PMC8230061 34072117

[88] LeMay-Nedjelski L., Yonemitsu C., Asbury M.R., Butcher J., Ley S.H., Hanley A.J. (2021). Oligosaccharides and microbiota in human milk are interrelated at 3 months postpartum in a cohort of women with a high prevalence of gestational impaired glucose tolerance. J. Nutr..

[89] Cabrera-Rubio R., Kunz C., Rudloff S., García-Mantrana I., Crehuá-Gaudiza E., Martínez-Costa C. (2019). Association of maternal secretor status and human milk oligosaccharides with milk microbiota: an observational pilot study. J. Pediatr. Gastroenterol. Nutr..

[90] Stinson L.F., Trevenen M.L., Geddes D.T. (2021). The viable microbiome of human milk differs from the metataxonomic profile. Nutrients.

[91] Gale C., Logan K.M., Santhakumaran S., Parkinson J.R., Hyde M.J., Modi N. (2012). Effect of breastfeeding compared with formula feeding on infant body composition: a systematic review and meta-analysis. Am. J. Clin. Nutr..

[92] Patro-Gołąb B., Zalewski B.M., Kouwenhoven S.M., Karaś J., Koletzko B., Bernard van Goudoever J. (2016). Protein concentration in milk formula, growth, and later risk of obesity: A systematic review. J. Nutr..

[93] Patro-Gołąb B., Zalewski B.M., Polaczek A., Szajewska H. (2019). Duration of breastfeeding and early growth: A systematic review of current evidence. Breastfeed. Med..

[94] Bell K.A., Wagner C.L., Feldman H.A., Shypailo R.J., Belfort M.B. (2017). Associations of infant feeding with trajectories of body composition and growth. Am. J. Clin. Nutr..

[95] U.S. Department of Agriculture, U.S. Department of Health and Human Services (2020). https://www.dietaryguidelines.gov/sites/default/files/2021-03/Dietary_Guidelines_for_Americans-2020-2025.pdf.

[96] Dewey K.G., Güngör D., Donovan S.M., Madan E.M., Venkatramanan S., Davis T.A. (2021). Breastfeeding and risk of overweight in childhood and beyond: a systematic review with emphasis on sibling-pair and intervention studies. Am. J. Clin. Nutr..

[97] Kramer M.S., Chalmers B., Hodnett E.D., Sevkovskaya Z., Dzikovich I., Shapiro S. (2001). Promotion of Breastfeeding Intervention Trial (PROBIT): a randomized trial in the Republic of Belarus. JAMA.

[98] Kramer M.S., Matush L., Vanilovich I., Platt R.W., Bogdanovich N., Sevkovskaya Z. (2007). Effects of prolonged and exclusive breastfeeding on child height, weight, adiposity, and blood pressure at age 6.5 y: evidence from a large randomized trial. Am. J. Clin. Nutr..

[99] Martens P.J. (2012). What do Kramer's Baby-friendly hospital initiative PROBIT studies tell us? A review of a decade of research. J. Hum. Lact..

[100] M.B. Azad, L. Vehling, D. Chan, A. Klopp, N.C. Nickel, J.M. McGavock, et al, Infant feeding and weight gain: separating breast milk from breastfeeding and formula from food. Pediatrics 142(4), 10.1542/peds.2018-1092. 30249624

[101] Gridneva Z., Rea A., Hepworth A.R., Ward L.C., Lai C.T., Hartmann P.E. (2018). Relationships between breastfeeding patterns and maternal and infant body composition over the first 12 months of lactation. Nutrients.

[102] A. Mazzocchi, M.L. Giannì, D. Morniroli, L. Leone, P. Roggero, C. Agostoni, et al., Hormones in breast milk and effect on infants' growth: A systematic review. Nutrients 11(8), 10.3390/nu11081845. PMC6724322 31395844

[103] Park J.J.H., Siden E., Harari O., Dron L., Mazoub R., Jeziorska V. (2019). Interventions to improve linear growth during exclusive breastfeeding life-stage for children aged 0-6 months living in low- and middle-income countries: a systematic review with network and pairwise meta-analyses. Gates Open Res.

[104] Dewey K.G., Lönnerdal B. (1986). Infant self-regulation of breast milk intake. Acta Paediatr. Scand..

[105] Timby N., Hernell O., Lönnerdal B., Domellöf M. (2014). Parental feeding control in relation to feeding mode and growth pattern during early infancy. Acta Paediatr.

[106] Williams J.E., McGuire M.K., Meehan C.L., McGuire M.A., Brooker S.L., Kamau-Mbuthia E.W. (2021). Key genetic variants associated with variation of milk oligosaccharides from diverse human populations. Genomics.

[107] Larsson M.W., Lind M.V., Laursen R.P., Yonemitsu C., Larnkjær A., Mølgaard C. (2019). Human milk oligosaccharide composition is associated with excessive weight gain during exclusive breastfeeding-an explorative study. Front. Pediatr..

[108] Alderete T.L., Autran C., Brekke B.E., Knight R., Bode L., Goran M.I. (2015). Associations between human milk oligosaccharides and infant body composition in the first 6 mo of life. Am. J. Clin. Nutr..

[109] A.S. Cheema, Z. Gridneva, A.J. Furst, A.S. Roman, M.L. Trevenen, B.A. Turlach, et al, Human milk oligosaccharides and bacterial profile modulate infant body composition during exclusive breastfeeding. Int. J. Mol. Sci. 23(5), 10.3390/ijms23052865. PMC8911220 35270006

[110] Georgieff M.K., Ramel S.E., Cusick S.E. (2018). Nutritional influences on brain development. Acta Paediatr.

[111] Anderson J.W., Johnstone B.M., Remley D.T. (1999). Breast-feeding and cognitive development: a meta-analysis. Am. J. Clin. Nutr..

[112] Andres A., Cleves M.A., Bellando J.B., Pivik R.T., Casey P.H., Badger T.M. (2012). Developmental status of 1-year-old infants fed breast milk, cow's milk formula, or soy formula. Pediatrics.

[113] Sajjad A., Tharner A., Kiefte-de Jong J.C., Jaddoe V.V., Hofman A., Verhulst F.C. (2015). Breastfeeding duration and non-verbal IQ in children. J. Epidemiol. Community Health.

[114] Jacobson S.W., Jacobson J.L. (2002). Breastfeeding and IQ: evaluation of the socio-environmental confounders. Acta Paediatr.

[115] Horta B.L., Victora C.G. (2013). World Health Organization.

[116] de Santa Barbara P., van den Brink G.R., Roberts D.J. (2003). Development and differentiation of the intestinal epithelium. Cell. Mol. Life Sci..

[117] Cummins A.G., Thompson F.M. (1997). Postnatal changes in mucosal immune response: a physiological perspective of breast feeding and weaning. Immunol. Cell Biol..

[118] Donovan S.M. (2006). Role of human milk components in gastrointestinal development: current knowledge and future needs. J. Pediatr..

[119] Donovan S.M., Odle J. (1994). Growth factors in milk as mediators of infant development. Annu. Rev. Nutr..

[120] Manners M.J., Stevens J.A. (1972). Changes from birth to maturity in the pattern of distribution of lactase and sucrase activity in the mucosa of the small intestine of pigs. Br. J. Nutr..

[121] Neu J. (2007). Gastrointestinal maturation and implications for infant feeding. Early Hum. Dev..

[122] Cacho N.T., Lawrence R.M. (2017). Innate immunity and breast milk. Front. Immunol..

[123] Dawod B., Marshall J.S., Azad M.B. (2021). Breastfeeding and the developmental origins of mucosal immunity: how human milk shapes the innate and adaptive mucosal immune systems. Curr. Opin. Gastroenterol..

[124] S.K. Dogra, C. Kwong Chung, D. Wang, O. Sakwinska, S. Colombo Mottaz, N. Sprenger, Nurturing the early life gut microbiome and immune maturation for long term health. Microorganisms 9(10), 10.3390/microorganisms9102110. PMC8537230 34683431

[125] Henrick B.M., Rodriguez L., Lakshmikanth T., Pou C., Henckel E., Arzoomand A. (2021). Bifidobacteria-mediated immune system imprinting early in life. Cell.

[126] Ardeshir A., Narayan N.R., Méndez-Lagares G., Lu D., Rauch M., Huang Y. (2014). Breast-fed and bottle-fed infant rhesus macaques develop distinct gut microbiotas and immune systems. Sci. Transl. Med..

[127] Andersson Y., Hammarström M.L., Lönnerdal B., Graverholt G., Fält H., Hernell O. (2009). Formula feeding skews immune cell composition toward adaptive immunity compared to breastfeeding. J. Immunol..

[128] Kramer M.S., Matush L., Vanilovich I., Platt R., Bogdanovich N., Sevkovskaya Z. (2007). Effect of prolonged and exclusive breast feeding on risk of allergy and asthma: cluster randomised trial. BMJ.

[129] Tannock G.W. (2021). Building robust assemblages of bacteria in the human gut in early life. Appl. Environ. Microbiol..

[130] Vandenplas Y., Carnielli V.P., Ksiazyk J., Luna M.S., Migacheva N., Mosselmans J.M. (2020). Factors affecting early-life intestinal microbiota development. Nutrition.

[131] L. Ye, J.F. Rawls, Microbial influences on gut development and gut-brain communication. Development 148 (21), 10.1242/dev.194936. PMC8627602 34758081

[132] Y.Y. Chen, X. Zhao, W. Moeder, H.M. Tun, E. Simons, P.J. Mandhane, et al, Impact of maternal intrapartum antibiotics, and Caesarean section with and without labour on Bifidobacterium and other infant gut microbiota. Microorganisms 9 (9), 10.3390/microorganisms9091847. PMC8467529 34576741

[133] Korpela K. (2021). Impact of delivery mode on infant gut microbiota. Ann. Nutr. Metab..

[134] Princisval L., Rebelo F., Williams B.L., Coimbra A.C., Crovesy L., Ferreira A.L. (2021). Association between the mode of delivery and infant gut microbiota composition UP to 6 months of age: A systematic literature review considering the role of breastfeeding. Nutr. Rev..

[135] O. Hernell, Human milk vs. cow's milk and the evolution of infant formulas (2011).

[136] Vandenplas Y., Berger B., Carnielli V.P., Ksiazyk J., Lagström N., Sanchez Luna M. (2018). Human milk oligosaccharides: 2′-fucosyllactose (2′-FL) and lacto-N-neotetraose (LNnT) in infant formula. Nutrients.

[137] Marriage B.J., Buck R.H., Goehring K.C., Oliver J.S., Williams J.A. (2015). Infants fed a lower calorie formula with 2'FL show growth and 2'FL uptake like breast-fed infants. J. Pediatr. Gastroenterol. Nutr..

[138] Goehring K.C., Marriage B.J., Oliver J.S., Wilder J.A., Barrett E.G., Buck R.H. (2016). Similar to those who are breastfed, infants fed a formula containing 2'-fucosyllactose have lower inflammatory cytokines in a randomized controlled trial. J. Nutr..

[139] Puccio G., Alliet P., Cajozzo C., Janssens E., Corsello G., Sprenger N. (2017). Effects of infant formula with human milk oligosaccharides on growth and morbidity: A randomized multicenter trial. J. Pediatr. Gastroenterol. Nutr..

[140] Dogra S.K., Martin F.P., Donnicola D., Julita M., Berger B., Sprenger N. (2021). Human milk oligosaccharide-stimulated Bifidobacterium species contribute to prevent later respiratory tract infections. Microorganisms.

[141] K. Parschat, C. Melsaether, K.R. Jäpelt, S. Jennewein, Clinical evaluation of 16-week supplementation with 5HMO-mix in healthy-term human infants to determine tolerability, safety, and effect on growth. Nutrients 13 (8), 10.3390/nu13082871. PMC8401119 34445031

[142] Lönnerdal B. (2009). Nutritional roles of lactoferrin. Curr. Opin. Clin. Nutr. Metab. Care.

[143] Lönnerdal B., Jiang R., Du X. (2011). Bovine lactoferrin can be taken up by the human intestinal lactoferrin receptor and exert bioactivities. J. Pediatr. Gastroenterol. Nutr..

[144] Shan T., Wang Y., Wang Y., Liu J., Xu Z. (2007). Effect of dietary lactoferrin on the immune functions and serum iron level of weanling piglets. J. Anim. Sci..

[145] Tomita M., Wakabayashi H., Shin K., Yamauchi K., Yaeshima T., Iwatsuki K. (2009). Twenty-five years of research on bovine lactoferrin applications. Biochimie.

[146] King J.C., Cummings G.E., Guo N., Trivedi L., Readmond B.X., Keane V. (2007). A double-blind, placebo-controlled, pilot study of bovine lactoferrin supplementation in bottle-fed infants. J. Pediatr. Gastroenterol. Nutr..

[147] Nguyen N.D., Wang D. (2020). Multiview learning for understanding functional multiomics. PLOS Comput. Biol..

[148] Chen K., Chai L., Li H., Zhang Y., Xie H.M., Shang J. (2016). Effect of bovine lactoferrin from iron-fortified formulas on diarrhea and respiratory tract infections of weaned infants in a randomized controlled trial. Nutrition.

[149] Hernell O., Lönnerdal B. (2002). Iron status of infants fed low-iron formula: no effect of added bovine lactoferrin or nucleotides. Am. J. Clin. Nutr..

[150] Manfredi M., Bizzarri B., Sacchero R.I., Maccari S., Calabrese L., Fabbian F. (2012). Helicobacter pylori infection in clinical practice: probiotics and a combination of probiotics + lactoferrin improve compliance, but not eradication, in sequential therapy. Helicobacter.

[151] Manzoni P., Meyer M., Stolfi I., Rinaldi M., Cattani S., Pugni L. (2014). Bovine lactoferrin supplementation for prevention of necrotizing enterocolitis in very-low-birth-weight neonates: a randomized clinical trial. Early Hum. Dev..

[152] Manzoni P., Rinaldi M., Cattani S., Pugni L., Romeo M.G., Messner H. (2009). Bovine lactoferrin supplementation for prevention of late-onset sepsis in very low-birth-weight neonates: a randomized trial. JAMA.

[153] ELFIN trial investigators group (2019). Enteral lactoferrin supplementation for very preterm infants: a randomised placebo-controlled trial. Lancet.

[154] Ochoa T.J., Zegarra J., Bellomo S., Carcamo C.P., Cam L., Castañeda A. (2020). Randomized controlled trial of bovine lactoferrin for prevention of sepsis and neurodevelopment impairment in infants weighing less than 2000 grams. J. Pediatr..

[155] Lonnerdal B., Du X., Jiang R. (2021). Biological activities of commercial bovine lactoferrin sources. Biochem Cell Biol.

[156] Li F., Wu S.S., Berseth C.L., Harris C.L., Richards J.D., Wampler J.L. (2019). Improved neurodevelopmental outcomes associated with bovine milk fat globule membrane and lactoferrin in infant formula: A randomized, controlled trial. J. Pediatr..

[157] Brink L.R., Brink L.R., Herren A.W., McMillen S., Fraser K., Agnew M., Roy N. (2020). Omics analysis reveals variations among commercial sources of bovine milk fat globule membrane. J. Dairy Sci..

[158] Zavaleta N., Zavaleta N., Kvistgaard A.S., Graverholt G., Respicio G., Guija H., Valencia N. (2011). Efficacy of an MFGM-enriched complementary food in diarrhea, anemia, and micronutrient status in infants. J. Pediatr. Gastroenterol. Nutr..

[159] Timby N., Timby N., Domellöf E., Hernell O., Lönnerdal B., Domellöf M. (2014). Neurodevelopment, nutrition, and growth until 12 mo of age in infants fed a low-energy, low-protein formula supplemented with bovine milk fat globule membranes: a randomized controlled trial. Am. J. Clin. Nutr.

[160] Timby N., Hernell O., Vaarala O., Melin M., Lönnerdal B., Domellöf M. (2015). Infections in infants fed formula supplemented with bovine milk fat globule membranes. J. Pediatr. Gastroenterol. Nutr..

[161] Timby N., Domellöf M., Holgerson P.L., West C.E., Lönnerdal B., Hernell O. (2017). Oral microbiota in infants fed a formula supplemented with bovine milk fat globule membranes - A randomized controlled trial. PLoS One.

[162] Grip T., Dyrlund T.S., Ahonen L., Domellöf M., Hernell O., Hyötyläinen T. (2018). Serum, plasma and erythrocyte membrane lipidomes in infants fed formula supplemented with bovine milk fat globule membranes. Pediatr. Res..

[163] Timby N., Adamsson M., Domellöf E., Grip T., Hernell O., Lönnerdal B. (2021). Neurodevelopment and growth until 6.5 years of infants who consumed a low-energy, low-protein formula supplemented with bovine milk fat globule membranes: a randomized controlled trial. Am. J. Clin. Nutr..

[164] Schack L., Lange A., Kelsen J., Agnholt J., Christensen B., Petersen T.E. (2009). Considerable variation in the concentration of osteopontin in human milk, bovine milk, and Infant Formulas. J. Dairy Sci..

[165] Jiang R., Lönnerdal B. (2019). Osteopontin in human milk and infant formula affects infant plasma osteopontin concentrations. Pediatr. Res..

[166] A. Aksan, I. Erdal, S.S. Yalcin, J. Stein, G. Samur, Osteopontin levels in human milk are related to maternal nutrition and infant health and growth. Nutrients 13(8) (2021) 2670, 10.3390/nu13082670. PMC8402120 34444830

[167] Lönnerdal B., Kvistgaard A.S., Peerson J.M., Donovan SM S.M., Peng Y.M. (2016). Growth, nutrition, and cytokine response of breast-fed infants and infants fed formula with added bovine osteopontin. J Pediatr Gastroenterol Nutr.

[168] West C.E., Kvistgaard A.S., Peerson J.M., Donovan S.M., Peng Y.M., Lönnerdal B. (2017). Effects of osteopontin-enriched formula on lymphocyte subsets in the first 6 months of life: a randomized controlled trial. Pediatr. Res..

[169] Liu L., Jiang R., Lönnerdal B. (2019). Assessment of bioactivities of the human milk lactoferrin-osteopontin complex in vitro. J. Nutr. Biochem..

[170] Harris W.S., Connor W.E., Lindsey S. (1984). Will dietary omega-3 fatty acids change the composition of human milk?. Am. J. Clin. Nutr..

[171] Sanders T.A., Naismith D.J. (1979). A comparison of the influence of breast-feeding and bottle-feeding on the fatty acid composition of the erythrocytes. Br. J. Nutr..

[172] Putnam J.C., Carlson S.E., DeVoe P.W., Barness L.A. (1982). The effect of variations in dietary fatty acids on the fatty acid composition of erythrocyte phosphatidylcholine and phosphatidylethanolamine in human infants. Am. J. Clin. Nutr..

[173] Benolken R.M., Anderson R.E., Wheeler T.G. (1973). Membrane fatty acids associated with the electrical response in visual excitation. Science.

[174] Neuringer M., Connor W.E., Van Petten C., Barstad L. (1984). Dietary omega-3 fatty acid deficiency and visual loss in infant rhesus monkeys. J. Clin. Invest..

[175] Clandinin M.T., Chappell J.E., Leong S., Heim T., Swyer P.R., Chance G.W. (1980). Intrauterine fatty acid accretion rates in human brain: implications for fatty acid requirements. Early Hum. Dev..

[176] Clandinin M.T., Chappell J.E., Leong S., Heim T., Swyer P.R., Chance G.W. (1980). Extrauterine fatty acid accretion in infant brain: implications for fatty acid requirements. Early Hum. Dev..

[177] Verfuerden M.L., Dib S., Jerrim J., Fewtrell M., Gilbert R.E. (2020). Effect of long-chain polyunsaturated fatty acids in infant formula on long-term cognitive function in childhood: A systematic review and meta-analysis of randomised controlled trials. PLoS One.

[178] B. Jasani, K. Simmer, S.K. Patole, S.C. Rao, Long chain polyunsaturated fatty acid supplementation in infants born at term, Cochrane Database Syst. Rev. 3 (3), 10.1002/14651858.CD000376.pub4. PMC6464574 28281303

[179] K. Moon, S.C. Rao, S.M. Schulzke, S.K. Patole, K. Simmer, Longchain polyunsaturated fatty acid supplementation in preterm infants, Cochrane Database Syst. Rev. 12 (12), 10.1002/14651858.CD000375.pub5. PMC6463838 27995607

[180] Birch E.E., Carlson S.E., Hoffman D.R., Fitzgerald-Gustafson K.M., Fu V.L., Drover J.R. (2010). The DIAMOND (DHA Intake And Measurement Of Neural Development) Study: a double-masked, randomized controlled clinical trial of the maturation of infant visual acuity as a function of the dietary level of docosahexaenoic acid. Am. J. Clin. Nutr..

[181] Colombo J., Carlson S.E., Cheatham C.L., Shaddy D.J., Kerling E.H., Thodosoff J.M. (2013). Long-term effects of LCPUFA supplementation on childhood cognitive outcomes. Am. J. Clin. Nutr..

[182] K. Liao, B.D. McCandliss, S.E. Carlson, J. Colombo, D.J. Shaddy, E.H. Kerling, et al., Event-related potential differences in children supplemented with long-chain polyunsaturated fatty acids during infancy, Dev. Sci. 20 (5), 10.1111/desc.12455. PMC5392436 27747986

[183] Lepping R.J., Honea R.A., Martin L.E., Liao K., Choi I.Y., Lee P. (2019). Long-chain polyunsaturated fatty acid supplementation in the first year of life affects brain function, structure, and metabolism at age nine years. Dev. Psychobiol..

[184] Verfürden M.L., Gilbert R., Lucas A., Jerrim J., Fewtrell M. (2021). Effect of nutritionally modified infant formula on academic performance: linkage of seven dormant randomised controlled trials to national education data. BMJ.

[185] Groh-Wargo S., Jacobs J., Auestad N., O'Connor D.L., Moore J.J., Lerner E. (2005). Body composition in preterm infants who are fed long-chain polyunsaturated fatty acids: a prospective, randomized, controlled trial. Pediatr. Res..

[186] Currie L.M., Tolley E.A., Thodosoff J.M., Kerling E.H., Sullivan D.K., Colombo J. (2015). Long chain polyunsaturated fatty acid supplementation in infancy increases length- and weight-for-age but not BMI to 6 years when controlling for effects of maternal smoking. Prostaglandins Leukot. Essent. Fatty Acids.

[187] Adjibade M., Davisse-Paturet C., Bernard J.Y., Adel-Patient K., Divaret-Chauveau A., Lioret S. (2022). Enrichment of infant formula with long-chain polyunsaturated fatty acids and risk of infection and allergy in the nationwide ELFE birth cohort. Allergy.

[188] Bode L., Raman A.S., Murch S.H., Rollins N.C., Gordon J.I. (2020). Understanding the mother-breastmilk-infant "triad". Science.

[189] X. Phen. Available online: https://www.phenxtoolkit.org/.

[190] Biorepository MsMHMR (2022). https://mommysmilkresearch.org/.

[191] (MILC) MILC. Internet. Available online: https://www.milcresearch.com/.

[192] Covey S.R. (1989).

[193] Lackey K.A., Williams J.E., Meehan C.L., Zachek J.A., Benda E.D., Price W.J. (2019). What's normal? Microbiomes in human milk and infant feces are related to each other but vary geographically: the Inspire study. Front. Nutr..

[194] Dingess K.A., Valentine C.J., Ollberding N.J., Davidson B.S., Woo J.G., Summer S. (2017). Branched-chain fatty acid composition of human milk and the impact of maternal diet: the Global Exploration of Human Milk (GEHM) Study. Am. J. Clin. Nutr..

[195] Allen L.H., Hampel D., Shahab-Ferdows S., Andersson M., Barros E., Doel A.M. (2021). The mothers, infants, and lactation quality (MILQ) study: A multi-center collaboration. Curr. Dev. Nutr..

[196] C. Glaser, E. Lattka, P. Rzehak, C. Steer, B. Koletzko, Genetic variation in polyunsaturated fatty acid metabolism and its potential relevance for human development and health. Matern. Child Nutr. (Suppl 2):27-40, 10.1111/j.1740-8709.2011.00319.x. PMC6860603 21366865

[197] Kunz C., Meyer C., Collado M.C., Geiger L., García-Mantrana I., Bertua-Ríos B. (2017). Influence of gestational age, secretor, and Lewis blood group status on the oligosaccharide content of human milk. J. Pediatr. Gastroenterol. Nutr..

[198] Internet. Available online: https://strapi-lactahub.org/uploads/2021_EFBRI_Framework_and_Checklist_Lacta_Hub_45d31b1d0d.pdf (accessed on April 22, 2022).

[199] Azad M.B., Nickel N.C., Bode L., Brockway M., Brown A., Chambers C. (2021). Breastfeeding and the origins of health: interdisciplinary perspectives and priorities. Matern. Child Nutr..

[200] Cooke N.J., Hilton M.L., National Research Council (U.S.). Committee on the Science of Team Science (2015).

[201] Azad M.B., Atkinson S., Geddes D., Edtion, McGuire M.K., O’Connor D.L. (2021).

[202] Hu D., Wang F., Zhang H., Wu Z., Zhou Z., Li G. (2022). Existence of Functional Connectome Fingerprint during Infancy and Its Stability over Months. J. Neurosci..

[203] Woodburn M., Bricken C.L., Wu Z., Li G., Wang L., Lin W. (2021). The maturation and cognitive relevance of structural brain network organization from early infancy to childhood. Neuroimage.

[204] Saben J.L., Sims C.R., Piccolo B.D., Andres A. (2020). Maternal adiposity alters the human milk metabolome: associations between nonglucose monosaccharides and infant adiposity. Am. J. Clin. Nutr..

[205] Sobik S., Sims C.R., McCorkle G., Bellando J., Sorensen S.T., Badger T.M. (2021). Early infant feeding effect on growth and body composition during the first 6 years and neurodevelopment at age 72 months. Pediatr. Res..

[206] Gallagher D., Andres A., Fields D.A., Evans W.J., Kuczmarski R., Lowe W.L. (2020). Body composition measurements from birth through 5 years: challenges, gaps, and existing & emerging technologies-A national institutes of health workshop. Obes. Rev..

[207] Samuel T.M., Zhou Q., Giuffrida F., Munblit D., Verhasselt V., Thakkar S.K. (2020). Nutritional and non-nutritional composition of human milk is modulated by maternal, infant, and methodological factors. Front. Nutr..

[208] McGuire M.K., Seppo A., Goga A., Buonsenso D., Collado M.C., Donovan S.M. (2021). Best practices for human milk collection for COVID-19 research. Breastfeed. Med..

[209] McGuire M.K., D.L O'Connor. Human Milk (2021).

[210] Hasin Y., Seldin M., Lusis A. (2017). Multi-omics approaches to disease. Genome Biol.

[211] C. Wu, F. Zhou, J. Ren, X. Li, Y. Jiang, S. Ma, A selective review of multi-level omics data integration using variable selection. High Throughput 8 (1), 10.3390/ht8010004. PMC6473252 30669303

[212] Yan J., Risacher S.L., Shen L., Saykin A.J. (2018). Network approaches to systems biology analysis of complex disease: integrative methods for multi-omics data. Brief. Bioinform..

[213] Lee B., Zhang S., Poleksic A., Xie L. (2019). Heterogeneous multi-layered network model for omics data integration and analysis. Front. Genet..

[214] Koh H.W.L., Fermin D., Vogel C., Choi K.P., Ewing R.M., Choi H. (2019). iOmicsPASS: network-based integration of multiomics data for predictive subnetwork discovery. NPJ Syst. Biol. Appl..

[215] Fang Z., Ma T., Tang G., Zhu L., Yan Q., Wang T. (2018). Bayesian integrative model for multi-omics data with missingness. Bioinformatics.

[216] Zhu L., Huo Z., Ma T., Oesterreich S., Tseng G.C. (2019). Bayesian indicator variable selection to incorporate hierarchical overlapping group structure in multi-omics applications. Ann. Appl. Stat..

[217] Chung N.C., Mirza B., Choi H., Wang J., Wang D., Ping P. (2019). Unsupervised classification of multi-omics data during cardiac remodeling using deep learning. Methods.

[218] Zhang L., Lv C., Jin Y., Cheng G., Fu Y., Yuan D. (2018). Deep learning-based multi-omics data integration reveals two prognostic subtypes in high-risk neuroblastoma. Front. Genet..

[219] Tarazona S., Balzano-Nogueira L., Gómez-Cabrero D., Schmidt A., Imhof A., Hankemeier T. (2020). Harmonization of quality metrics and power calculation in multi-omic studies. Nat. Commun..

[220] Krassowski M., Das V., Sahu S.K., Misra B.B. (2020). State of the Field in multi-omics research: from computational needs to data mining and sharing. Front. Genet..

[221] M. Love, Internet. Available online: https://github.com/mikelove/awesome-multi-omics (accessed on December 1, 2022).

[222] Ghaemi M.S., DiGiulio D.B., Contrepois K., Callahan B., Ngo T.T.M., Lee-McMullen B. (2019). Multiomics modeling of the immunome, transcriptome, microbiome, proteome and metabolome adaptations during human pregnancy. Bioinformatics.

[223] Jehan F., Sazawal S., Baqui A.H., Nisar M.I., Dhingra U., Khanam R. (2020). Multiomics characterization of preterm birth in low- and middle-income countries. JAMA Netw. Open.

[224] Marić I., Tsur A., Aghaeepour N., Montanari A., Stevenson D.K., Shaw G.M. (2020). Early prediction of preeclampsia via machine learning. Am. J. Obstet. Gynecol. MFM.

[225] Li E., Luo T., Wang Y. (2019). Identification of diagnostic biomarkers in patients with gestational diabetes mellitus based on transcriptome gene expression and methylation correlation analysis. Reprod. Biol. Endocrinol..

[226] Stelzer I.A., Ghaemi M.S., Han X., Ando K., Hédou J.J., Feyaerts D. (2021). Integrated trajectories of the maternal metabolome, proteome, and immunome predict labor onset. Sci. Transl. Med..

[227] López de Maturana E., Alonso L., Alarcón P., Martín-Antoniano I.A., Pineda S., Piorno L. (2019). Challenges in the integration of omics and non-omics data. Genes (Basel).

[228] Subramanian I., Verma S., Kumar S., Jere A., Anamika K. (2020). Multi-omics data integration, interpretation, and its application. Bioinform. Biol. Insights.

[229] Li Y., Wu F.X., Ngom A. (2018). A review on machine learning principles for multi-view biological data integration. Brief. Bioinform..

[230] Baltrusaitis T., Ahuja C., Morency L.P. (2019). Multimodal machine learning: A survey and taxonomy. I.E.E.E. Trans. Pattern Anal. Mach. Intell..

[231] J. Ngiam, https://ccrma.stanford.edu/∼juhan/pubs/NgiamKhoslaKimNamLeeNg10.pdf (accessed April 22, 2022).

[232] Zhang Y., Yang Q. (2021). A survey on multi-task learning. I.E.E.E. Trans. Knowl. Data Eng..

[233] Caruana R. (1997). Multitask learning. Mach. Learn..

[234] Ruder S. (2017).

[235] Harutyunyan H., Khachatrian H., Kale D.C., Ver Steeg G., Galstyan A. (2019). Multitask learning and benchmarking with clinical time series data. Sci. Data.

[236] Li Y., Yang M., Zhang Z. (2019). A survey of multi-view representation learning. I.E.E.E. Trans. Knowl. Data Eng..

[237] Shenhav L., Azad M.B. (2022). Using community ecology theory and computational microbiome methods to study human milk as a biological system. mSystems.

[238] L. Zheng, Y. Cheng, J. HeDeep multimodality model for multi-task multi-view learning. In: Edtion, ed. Proceedings of the 2019 SIAM International Conference on Data Mining, SDM Press; 10-18.

[239] Seppo A.E., Choudhury R., Pizzarello C., Palli R., Fridy S., Rajani P.S. (2021). Traditional farming lifestyle in old older Mennonites modulates human milk composition. Front. Immunol..

[240] Fernández L., Pannaraj P.S., Rautava S., Rodríguez J.M. (2020). The microbiota of the human mammary ecosystem. Front. Cell. Infect. Microbiol..

[241] Pannaraj P.S., Ly M., Cerini C., Saavedra M., Aldrovandi G.M., Saboory A.A. (2018). Shared and distinct features of human milk and infant stool viromes. Front. Microbiol..

[242] Murphy K., Curley D., O'Callaghan T.F., O'Shea C.A., Dempsey E.M., O'Toole P.W. (2017). The composition of human milk and infant faecal microbiota over the first three months of life: A pilot study. Sci. Rep..

[243] Reyes S.M., Allen D.L., Williams J.E., McGuire M.A., McGuire M.K., Hay A.G. (2021). Pumping supplies alter the microbiome of pumped human milk: an in-home, randomized, crossover trial. Am. J. Clin. Nutr..

[244] Fehr K., Moossavi S., Sbihi H., Boutin R.C.T., Bode L., Robertson B. (2020). Breastmilk feeding practices are associated with the co-occurrence of bacteria in mothers' milk and the infant gut: the CHILD cohort study. Cell Host Microbe.

[245] Seppo A.E., Bu K., Jumabaeva M., Thakar J., Choudhury R.A., Yonemitsu C. (2021). Infant gut microbiome is enriched with Bifidobacterium longum ssp. infantis in old order Mennonites with traditional farming lifestyle. Allergy.

